# SEE: A Method for Predicting the Dynamics of Chromatin Conformation Based on Single‐Cell Gene Expression

**DOI:** 10.1002/advs.202406413

**Published:** 2025-01-07

**Authors:** Minghong Li, Yurong Yang, Rucheng Wu, Haiyan Gong, Zan Yuan, Jixin Wang, Erping Long, Xiaotong Zhang, Yang Chen

**Affiliations:** ^1^ State Key Laboratory of Common Mechanism Research for Major Diseases Department of Biochemistry and Molecular Biology Institute of Basic Medical Sciences Chinese Academy of Medical Sciences and Peking Union Medical College Beijing 100005 China; ^2^ Department of Computer Science and Technology University of Science and Technology Beijing Beijing 100083 China; ^3^ Beijing Advanced Innovation Center for Materials Genome Engineering University of Science and Technology Beijing Beijing 100083 China; ^4^ School of Basic Medicine Tsinghua University Beijing 100084 China; ^5^ The State Key Laboratory of Respiratory Health and Multimorbidity Institute of Basic Medical Sciences Chinese Academy of Medical Sciences and Peking Union Medical College Beijing 100005 China; ^6^ Shunde Innovation School University of Science and Technology Beijing Foshan 528399 China

**Keywords:** chromatin dynamics, single cell Hi‐C, single cell RNA‐seq

## Abstract

The dynamics of chromatin conformation involve continuous and reversible changes within the nucleus of a cell, which participate in regulating processes such as gene expression, DNA replication, and damage repair. Here, SEE is introduced, an artificial intelligence (AI) method that utilizes autoencoder and transformer techniques to analyze chromatin dynamics using single‐cell RNA sequencing data and a limited number of single‐cell Hi‐C maps. SEE is employed to investigate chromatin dynamics across different scales, enabling the detection of (i) rearrangements in topologically associating domains (TADs), and (ii) oscillations in chromatin interactions at gene loci. Additionally, SEE facilitates the interpretation of disease‐associated single‐nucleotide polymorphisms (SNPs) by leveraging the dynamic features of chromatin conformation. Overall, SEE offers a single‐cell, high‐resolution approach to analyzing chromatin dynamics in both developmental and disease contexts.

## Graphical abstract

This study develops an artificial intelligence method, SEE, for analyzing chromatin dynamics in single cells, offering a high‐resolution and multi‐scale view of chromatin conformation changes. By detecting TAD rearrangement and defining dynamic index, SEE opens up new possibilities for understanding the regulatory mechanisms governing gene expression and chromatin organization in development and disease.

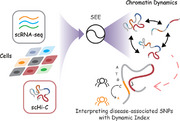

## 1 Introduction

High‐resolution analysis of chromatin conformation at spatial and temporal scales is crucial for a quantitative understanding of key molecular events within the nucleus.^[^ ^1^, ^2^ ^]^ This analysis forms the basis for precision gene therapies for diseases.^[^ ^3^ ^]^ While microscopy‐based methods provide direct measurements of time‐varying 3D distances between loci, they typically probe a limited number of loci.^[^ ^4^ ^]^ Single‐cell Hi‐C can effectively capture genome‐wide contact frequency maps but is hindered by low‐resolution and complex experimental procedures.^[^ ^5^ ^]^ Advanced in single‐cell multimodal omics has enabled the investigation of gene regulatory programs at unprecedented resolution and scale.^[^ ^5^, ^6^, ^7^, ^8^ ^]^ However, due to the typically high per‐cell cost, these technologies are costly for large‐scale analysis of complex heterogeneous samples and identification of rare cell states. Recently, generative AI methods, such as those based on autoencoders and transformers, have shown impressive results across various fields.^[^ ^9^ ^]^ Therefore, we introduce SEE, a single‐cell method that predicts single‐cell Hi‐C maps from single‐cell gene expression data using a transformer‐ and autoencoder‐based neural network.

SEE enables the characterization of chromatin conformation at the single‐cell level using single‐cell RNA‐seq data and a limited number of single‐cell Hi‐C maps. In our study, we quantitatively assess the accuracy of this method in predicting chromatin conformation and apply it to investigate chromatin dynamics across different scales, identifying rearrangements in topologically associating domains (TADs) and oscillations in chromatin interactions at gene loci. Furthermore, SEE aids in interpreting disease‐associated SNPs by characterizing regions related to *cis*‐regulatory elements using dynamic index.

## 2 Results

### 2.1 Overview of SEE

**Figure 1:**
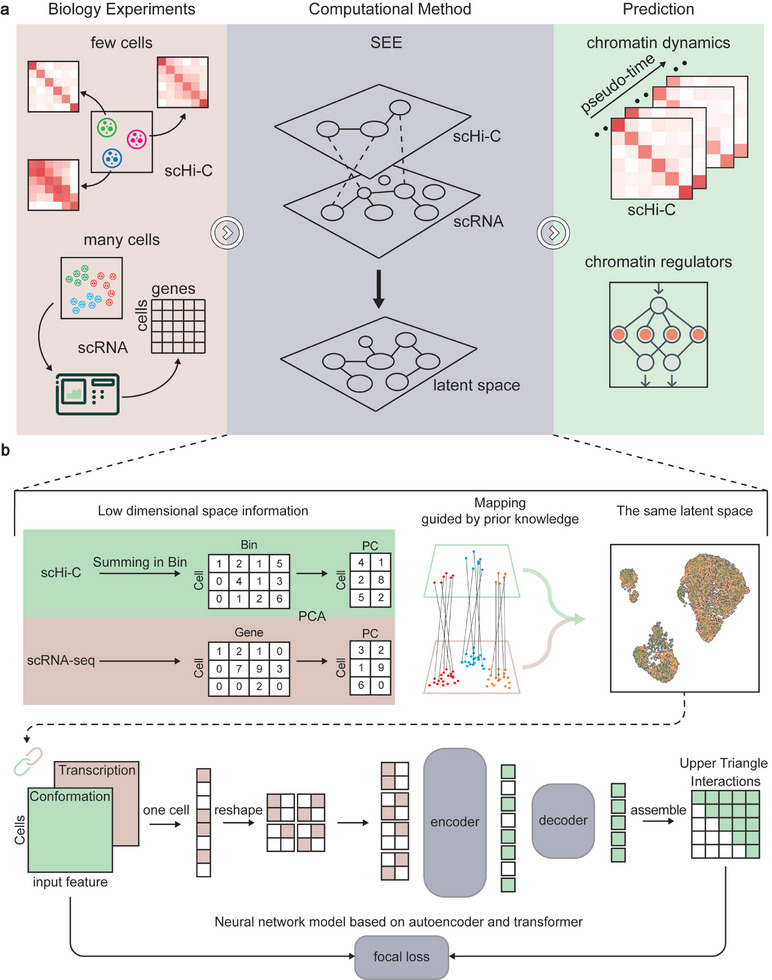
The usage scenarios and workflows of SEE. a) Training and application scenarios for SEE. The SEE framework utilizes the scRNA expression data and scHi‐C maps as input for training. Upon training, the model predicts the scHi‐C map of specific genomic regions based on the RNA expression data input. For downstream applications, the trained model can infer the chromatin dynamics and identify the chromatin regulators that affect the chromatin conformation in specific genomic regions. b) Framework of SEE. Up, scRNA samples and scHi‐C samples are integrated into the same low‐dimensional space through dimension reduction and association graph construction. The connectivity between the samples is determined by the distance in the common latent space. Down, the architecture of the SEE model. The input to the model is the scRNA data of one cell (1×N). The input vector is then converted to 2D matrix by reshape function. Then, the Encoder encodes the transformed data to obtain a set of latent vectors. Next, this latent vector is input into the Decoder to infer the corresponding position's scHi‐C upper triangular map data.

SEE is an architecture designed to predict contact frequency maps in specific genomic regions at the single‐cell level (**Figure** **1**a). SEE utilizes training data derived from single‐cell RNA (scRNA) expression data and single‐cell Hi‐C (scHi‐C) maps. During the training process, SEE takes scRNA‐seq data for all genes as input and outputs scHi‐C maps for a single specific region. After training with single‐cell data, SEE can predict scHi‐C maps for specific genomic regions solely based on RNA expression data. Furthermore, feature attribution analysis of the trained models can be employed to identify chromatin regulators.

The algorithm design of SEE involves two steps:

(1) Integration of cell embeddings for scRNA expression data and scHi‐C maps (Figure 1b up; Figure S1, Supporting Information). To model cell states, we normalized and scaled scRNA expression data and scHi‐C maps to obtain their respective low‐dimensional spatial information. Taking advantage of prior biological knowledge, we constructed the pseudo‐distance between the features of scRNA expression data and scHi‐C maps in graph representation. Specifically, the vertices are genes and chromatin regions (that is, 10‐kilobase (Kb) bins), and a positive edge will be connected between a gene and its genomic locations. Then, guided by the feature embeddings encoded from the graph, multimodal alignment as an iterative optimization procedure to map scRNA and scHi‐C data into the same low‐dimensional space.

We assessed the accuracy of the mapping on both paired and unpaired datasets (Figure 1b; Figure S2, Supporting Information). The evaluations with the HiRES dataset^[^ ^5^ ^]^ demonstrated that SEE can effectively distinguish between scHi‐C samples originating from various cell subtypes (Figure S2a, Supporting Information). These scHi‐C samples are initially mixed in the uniform manifold approximation and projection (UMAP) view.^[^ ^5^ ^]^ After mapping, 90.58% of the samples matched the actual cell subtype (Figure S2b, Supporting Information).

(2) Construction of a neural network model to learn the mapping of scRNA expression data to scHi‐C maps (Figure 1b down). We used a transformer as a concrete model for the encoder and decoder to learn the dependencies between features (gene expression) in low‐information‐density input data (scRNA). To enhance the performance of attention mechanisms in exploiting a large number of features, we transformed input data into a 2D matrix and embedded fixed‐size patches split from this matrix. Since the emphasis is on the initial information extraction for what affects the chromatin structure, a more complex encoder network is needed to extract information from input data. Because of the small amount of data to be predicted, a simple decoder model is sufficient to interpret the dimension reduction information after information extraction. Moreover, we used focal loss to mitigate class imbalance issues, which preserves the overall accuracy of mean square error (MSE) loss and yields improved training results in smaller‐size cell subtypes (astrocyte (Astro) and oligodendrocyte progenitor cell (OPC)) (Figure S3a,b, Supporting Information). Finally, SEE generates the upper triangle information of the scHi‐C contact matrix.

We systematically evaluated the performance of SEE across different metrics, locations, and scales. The evaluation of scHi‐C data showed that the large diagonal value will lead to bias in model training and correlation calculations (Figure S3c‐f, Supporting Information). We therefore excluded diagonal data when calculating similarity. Then, evaluations at multiple random regions showed that SEE can achieve robust performance at different scales and chromatin states (Table S1, Supporting Information; Experimental section). Notably, the evaluations across various scales revealed that large target Hi‐C map provides additional information and diverse features for the model, promoting its learning (Figure S3g, Supporting Information).

### 2.2 SEE Robustly Predicts Cell‐Specific Chromatin Conformation

**Figure 2:**
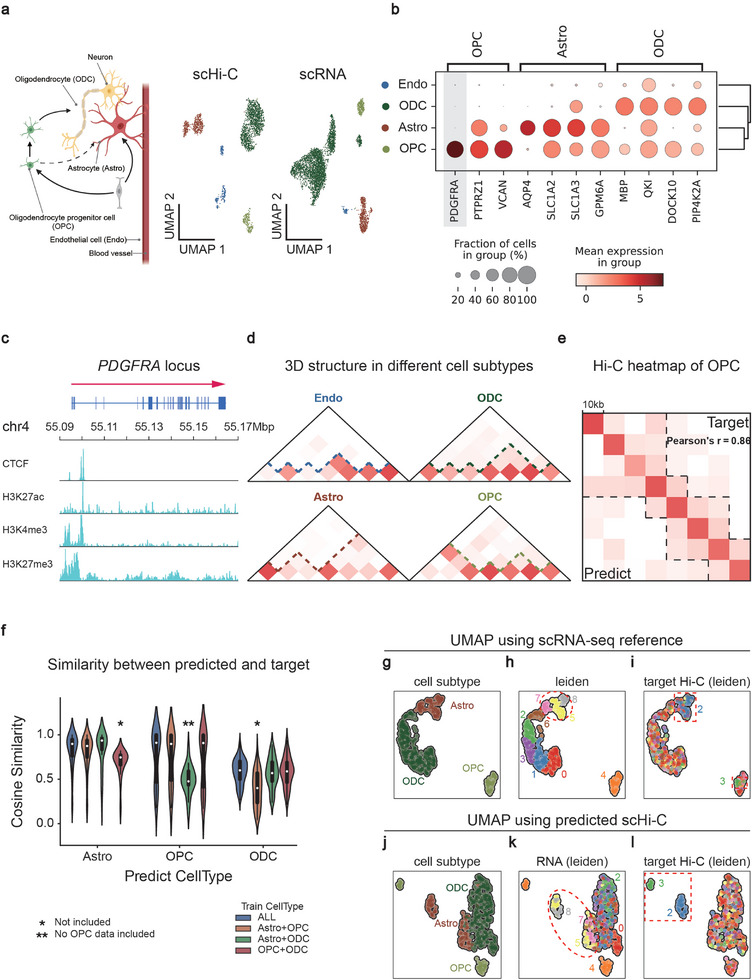
Robustness and benchmarking analysis for SEE. a) UMAP embedding of the human brain cortex partial cell subtypes identified in scHi‐C (Lee2019) and scRNA‐seq (M1‐10X GENOMICS) data. b) Cell fraction and mean expression of high differentially expressed genes in OPC, Astro, and ODC (M1‐10X GENOMICS dataset). c,d) The pseudo‐bulk Hi‐C map in *PDGFRA* locus (chr4: 55.09‐55.17Mb) of four cell subtypes (Astro, Endo, OPC, ODC) in the Lee2019 dataset. e) OPC‐pseudo‐bulk Hi‐C map of target and predicted in *PDGFRA* locus. f) Three cell subtypes (Astro, ODC, and OPC) cross‐validation comparison. Each violin shows the cosine similarity scores for target scHi‐C maps and predicted scHi‐C maps. g‐l) Reduced dimensional analysis of scRNAs and predicted scHi‐C maps in *PDGFRA* locus. First, use Principal Component Analysis (PCA) to reduce the features (interactions) of scHi‐C maps to ten dimensions, then calculate a neighborhood graph of observations using the Scanpy::neighbors function, and finally use UMAP for visualization. (**a** left) was created in BioRender. Li, M. (2024) https://BioRender.com/p85o497.

The unpaired human dataset from human brain cortex cells, containing scHi‐C (Lee2019^[^ ^10^ ^]^) and scRNA‐seq (Bakken et al.^[^ ^11^ ^]^) data, was used to evaluate the performance of SEE in developmental contexts (**Figure** **2**a). By comparing differential expression across cell subtypes, we identified widely reported marker genes, including oligodendrocyte progenitor cell (*PDGFRA*), oligodendrocyte (*MBP*, *MOG*), and astrocyte (*AQP4*, *SLC1A2*).

Furthermore, we noted that these differentially expressed genes (DEGs) exhibit heterogeneity in scRNA expression data and scHi‐C maps among various cell subtypes. Taking *PDGFRA* as an example, gene expression counts within multiple cell subtypes exhibited variations in the Bakken et al. dataset,^[^ ^11^ ^]^ which is evident in OPC (Figure 2b). Similarly, in the dataset provided by Lee2019,^[^ ^10^ ^]^ the scHi‐C map in the *PDGFRA* locus exhibited variations among the four cell subtypes (Figure 2c,d).

Our evaluations on the *PDGFRA* gene have demonstrated SEE's ability to distinguish heterogeneity among cell subtypes. Pseudo‐bulk Hi‐C maps were calculated using the target/predicted scHi‐C maps for different cell subtypes. We observed a significant correlation between SEE's OPC‐pseudo‐bulk Hi‐C map and the target's OPC‐pseudo‐bulk Hi‐C map (Figure 2e; Pearson's *r*  =  0.86, *P*  =  1.5×10^−11^ when not referring to diagonal data). Extensive evaluations under randomly selected 10 marker genes, 12 housekeeping genes, and 12 domain regions showed that SEE enable robust predict pseudo‐bulk Hi‐C map in different genomic regions (Table S1, Supporting Information; Experimental section). Expectedly, this correlation is identical to that observed at the single‐cell level (Figure 2f). We conducted dimensionality reduction analysis on the predicted scHi‐C maps. The results showed that different classes were distinguishable (Figure 2j).

To study whether our approach can generalize across cell types, we applied SEE to perform cross‐validation on all three cell subtypes. Since OPC is the characterized cell subtype of *PDGFRA*, the absence of OPC data during training will have a substantial impact on the model's predictive performance for this cell subtype (Figure 2f). It is noted that acquiring knowledge about oligodendrocyte (ODC) cell subtype proves relatively challenging, and inferred that the chromatin conformation of *PDGFRA* is disordered in ODC cell subtype (Figure 2f; Figure S4a, Supporting Information). Moreover, SEE can distinguish expression data of untrained cell subtypes and reflect in predicted results (Figure S4b, Supporting Information). Taken together, our results indicated that SEE can detect the differences between different cell stages.

Dimensionality reduction analysis illustrates SEE's capacity to adapt Hi‐C information based on RNA data. The distribution of Astro samples close to ODC in predicted scHi‐C maps is consistent with that in scRNA expression data (Figure 2g,h,j,k). Meanwhile, a subset of samples was isolated in predicted scHi‐C, and these samples were also clustered in the scRNA data (Figure 2i,l; Figure S5a,b, Supporting Information). We speculated that these samples had undergone complete differentiation, leading to chromatin conformation and expression heterogeneity. Moreover, we used the adjusted Rand index (ARI) to measure the clustering accuracy. The ARI values of the predicted local scHi‐C within *PDGFRA*, *MBP*, and *SLC1A3* ranged from 0.25 to 0.3. These values are slightly lower than the ARI of the original scHi‐C maps, which contain chromatin information across the entire genome (Figure S5c, Supporting Information).

We also examined whether there are factor‐binding motifs enriched in frequent interaction predicted by SEE. The predicted scHi‐C maps in the *SLC1A2* locus (Astro) were validated using CTCF data (obtained by WashU Epigenome Browser^[^ ^12^ ^]^) and alternative splicing events data (predicted by SUPPA2^[^ ^13^ ^]^). By analyzing the interactions and CTCF for each 10‐Kb bin, we found a positive correlation between them, consistent with the analysis of alternative splicing events (Figure S6, Supporting Information; CTCF Spearman's *ρ*  =  0.37, *P*  =  0.13; Splicing Spearman's *ρ*  =  0.43, *P*  =  0.074). The evaluations across multiple domains and genes also yielded the same conclusion (Figure S6c,d, Supporting Information). Previous studies revealed the link between CTCF binding/alternative splicing events and chromatin domain boundaries,^[^ ^14^, ^15^ ^]^ where more interactions are generally generated, and this fits with the analytical results.

Dozens of single‐cell samples contain the primary information of bulk Hi‐C. We calculated the Pearson correlation coefficients between the N‐sample‐pseudo‐bulk Hi‐C map and the two background Hi‐C maps in the *SLC1A2* locus (Astro) (Figure S7a,b, Supporting Information). As the sample size increases, Pearson's *r* of target/predicted would have a rapid climb and fluctuate greatly in the early stage, eventually converging within a similar range (spinal cord: 0.608 (target) and 0.593 (predicted); cerebellum: 0.446 (target) and 0.428 (predicted)). Notably, when the sample size exceeds 20, Pearson's *r* is close to the maximum. This suggests that 20‐sample‐pseudo‐bulk Hi‐C already contains most of the information from bulk Hi‐C (consistent with Figure 2d in ref.[16]). Quantitative results based on 10 random 1Mb length regions validated this conclusion (Figure S7c, Supporting Information). We also found that the Pearson's *r* of the spinal cord is higher than the cerebellum in *SLC1A2* locus (Astro) (Figure S7b,c, Supporting Information). This is possible because the main excitatory neurons in the brain and spinal cord are glutamatergic rather than GABAergic, and the protein encoded by *SLC1A2* is the primary carrier of glutamate.^[^ ^17^, ^18^ ^]^

All these results showed that SEE effectively captures subtle differences among cells in the input scRNA expression data and identifies correlated chromatin conformation patterns from the scHi‐C map. Consequently, SEE can make accurate and reliable predictions to reflect the variability in cell types and states.

### 2.3 SEE is Applicable to Different Species and Different Genes

To verify the transferability of the method, we performed experiments on the mice dataset (scHi‐C: Tan2021,^[^ ^19^ ^]^ scRNA: Allen Cell Types Database^[^ ^20^ ^]^) with the same steps. The results showed that scHi‐C and scRNA data were separated by different cell subtypes (Figure S8a, Supporting Information), and the predicted Hi‐C maps were similar to the observed results in *Slc1a2* and *Slc1a3* (Figures S8b and S9, Supporting Information. *Slc1a2* Pearson's *r*  =  0.837 (Astro), 0.802 (Micro), 0.319 (Oligo); *Slc1a3* Pearson's *r*  =  0.698 (Astro), 0.617 (Micro), 0.448 (Oligo); Pearson calculation does not refer to diagonal data). We further compared chromatin conformational differences near gene *Slc1a3*, which is widely considered as a marker gene for mouse astrocytes. The result showed that interaction signals increased by 3–10 times at enhancer‐promoter locations when using Astro cell subtype data to predict (Figure S9, Supporting Information).

### 2.4 SEE Observed TADs Rearrangement during Cell Differentiation

**Figure 3:**
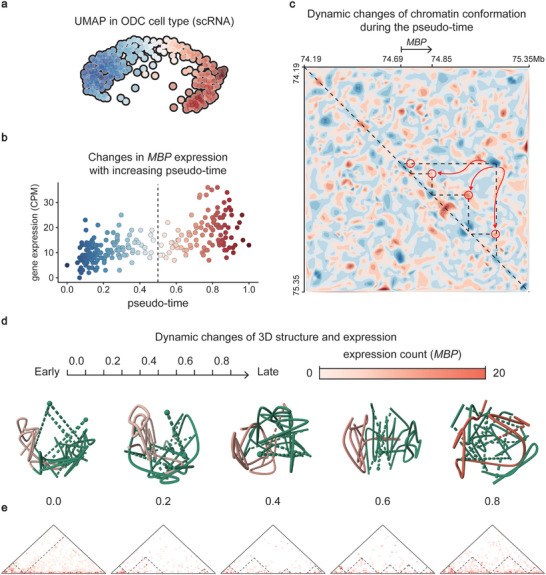
SEE identifies dynamic TADs boundary during the development of the ODC cell subtype. a‐c) and Figure S11 (Supporting Information) use the same coloring criterion (pseudo‐time), with specific indicators referring to the horizontal coordinates of b. a, UMAP representation of the Scanpy pseudo‐time fit shows trajectory of the ODC cells. b, Using the scRNA data used in a, calculate the association between *MBP* gene expression and pseudo‐time. c, Pseudotemporal dynamics of chromatin conformation near the *MBP* locus. Each scHi‐C map randomly takes several primary interactions using interaction values as probability. Then, the interaction density map is obtained by interpolation. (Experimental section) d) The 3D structure marked with *MBP* expression count (CPM). The scHi‐C data are analyzed based on their pseudo‐time, which is divided into ten‐time intervals ranging from “Early” to “Late”. The 3D structures were obtained by calculating the predicted pseudo‐bulk Hi‐C maps with 3DMax. **e**, The raw pseudo‐bulk Hi‐C of d.

To assess the relationship between chromatin conformation and cell differentiation, we jointly profile contact map and gene expression at a pseudo‐time scale (**Figure** **3**a–c; Figure S10 and Video S1, Supporting Information). Taking *MBP* as an example, the UMAP plot visually presents scRNA data from the ODC cell subtype as a distinct arc, displaying evident progressive connections during pseudo‐temporal analysis using Scanpy^[^ ^21^ ^]^ (Figure 3a). By examining gene expression values at various pseudo‐times, a clear upward trend in gene expression can be observed as pseudo‐time progresses, indicating that *MBP* expression changes along with the developmental process of ODC^[^ ^22^ ^]^ (Figure 3b).

The interaction density map revealed TAD rearrangements that regulate *MBP* expression in ODC. There is growing evidence suggests variability in the strength of TAD boundaries.^[^ ^23^, ^24^ ^]^ Upon extended observations near the *MBP* locus, we discovered that sub‐TAD structures undergo dynamic changes during ODC cell subtype differentiation (Figure 3c). As the pseudo‐time advanced, a large TAD was split into four smaller TADs, two of which were located within the *MBP* locus. We applied the same training and analysis approach to the *MBP* locus and reached consistent conclusions (Figure S11, Supporting Information). Moreover, the 3D reconstruction of the pseudo‐bulk Hi‐C within this region yielded similar observations: in the early pseudo‐time, all loci tend to aggregate and be continuous, while in the late pseudo‐time, they are divided into four contact domains (Figure 3d,e). This discovery implies that sub‐TAD boundaries near the *MBP* locus are not rigid, and their plasticity is associated with changes in gene expression during differentiation.

### 2.5 SEE Unveils the Oscillation of Chromatin Interactions at the Gene Locus

**Figure 4:**
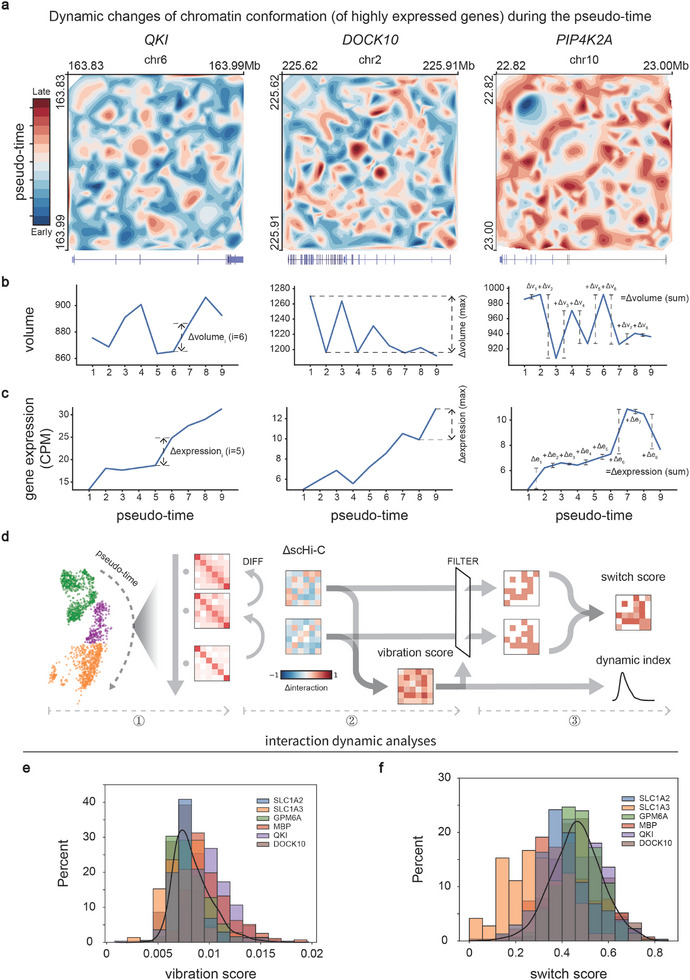
SEE enables detailed characterization of gene‐locus chromatin oscillations. a) Differences in changes of Hi‐C maps for three genes (*QKI*, *DOCK10*, *PIP4K2A*) locus under the pseudo‐time scale. b) The chromatin conformation volume changes over pseudo‐time. The volume is defined as the volume of the convex hull,^[^ ^55^ ^]^ which composed of 3D point set derived from 3DMax. c) The expression count (CPM) changes over pseudo‐time. d) Analytical workflow of interaction dynamics (Methods). e and f, Histogram of the vibration score and the switch score for interactions. e,f) are plotted with multiple genes (*SLC1A2*, *SLC1A3*, *GPM6A*, *MBP*, *QKI*, and *DOCK10*) and cell subtypes (Astro and ODC).

We further analyzed the scHi‐C maps changes of multiple genes at the pseudo‐time scale and found that their patterns of interaction migration differed. Taking the marker genes related to the ODC cell subtype as an example, the interaction of the *QKI* locus is dispersed at the early stage, while the *DOCK10* locus is relatively stable, and the *PIP4K2A* locus is the opposite (**Figure** **4**a). This change promotes the proximity of designated chromosomal loci, thereby upregulating gene expression.

To quantitatively characterize the changes in chromatin conformation within the pseudo‐time process, we reconstructed the 3D structure^[^ ^25^ ^]^ and calculated their volumes. After defining the distribution of volume and its changes, we observed that extensive chromatin oscillations occur at the gene locus, which resembles a switch driven by chromatin dynamics (Figure 4b; Figure S12a, Supporting Information; Experimental Section). Moreover, we observed that the average Δvolume approached almost zero (Figure S12c, Supporting Information). This suggests that the change in chromatin conformation volume across the entire pseudo‐time scale is not directly related to gene expression (Figure 4b,c; Figure S12a,b, Supporting Information).

**Figure 5:**
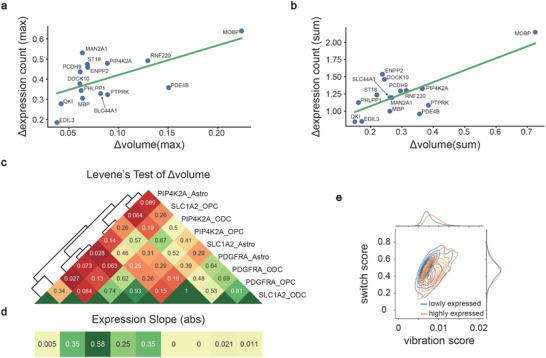
Analysis of the chromatin dynamics and gene expression dynamics. a‐b) Correlation analysis of oscillation patterns in gene expression counts and chromatin conformation volume on a pseudo‐time scale. c) Heatmap representation of variance similarity for Δvolume distribution. d) The slope of gene expression during the development of their cell subtype in c. e) The dynamic score density estimation for marker genes in cell subtypes with high expression levels (Astro: *SLC1A2*, *SLC1A3*, and *GPM6A*; ODC: *MBP*, *QKI*, and *DOCK10*) and those with low expression levels (Astro: *MBP*, *QKI*, and *DOCK10*; ODC: *SLC1A2*, *SLC1A3*, and *GPM6A*).

**Figure 6:**
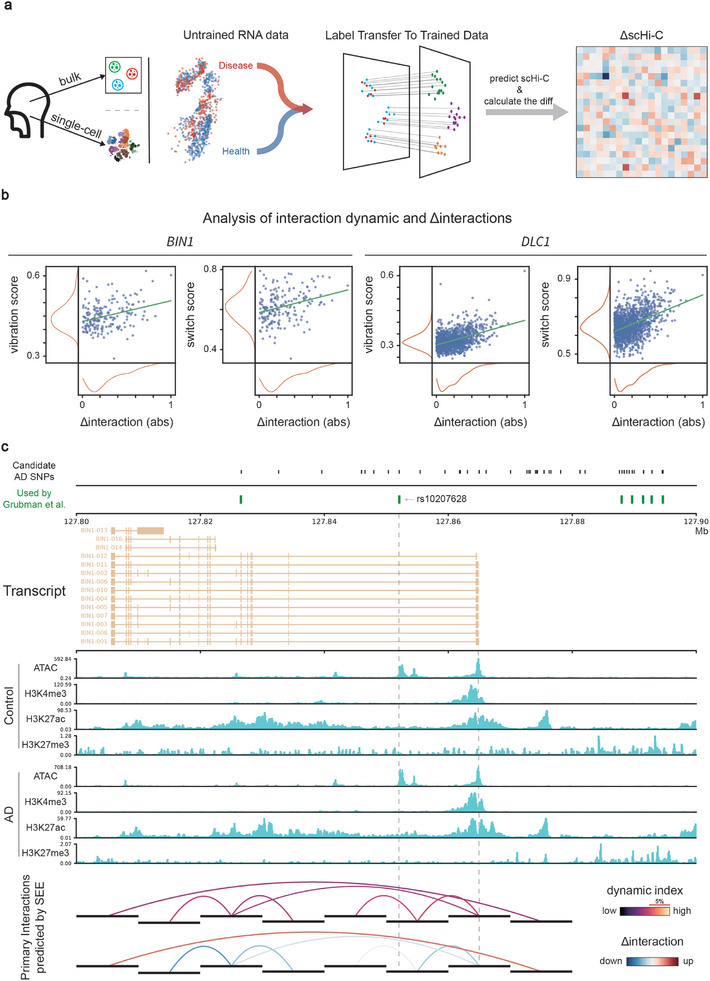
Application of SEE in Alzheimer's disease. a) General usage of SEE to analyze untrained RNA data. Use scanorama to map the untrained and trained scRNA data into the same space and then transfer the labels back to the trained scRNA data. Next, SEE uses scRNA data to predict the scHi‐C map and then calculates the difference in the scHi‐C map between the disease and health samples (ΔscHi‐C). b) Correlation analysis of the dynamic score with the Δinteraction within *BIN1* and *DLC1* locus. Δinteraction represents the difference in interaction value between disease and health states. c) SEE‐identified dynamic chromatin interactions from astrocyte cell subtype around gene *BIN1*. Candidate AD SNPs are the SNPs in high LD (r2>0.8) with lead SNPs reported in AD GWAS.

To investigate the intrinsic dynamic of chromatin conformation, we proposed vibration score and switch score to characterize interaction dynamics (Figure 4d). As the threshold for determining whether interactions oscillate is sample‐specific, switch score is suitable for assessing dynamic variations related to biological events within the same sample (Figure 4e,f; Figure 6b; Figure S13, Supporting Information). Vibration score is designed for assessing dynamic variations across multiple samples, aiding in the identification of heterogeneity in chromatin dynamics. Utilizing the vibration score, we observed that interactions of marker genes in cell subtypes with high expression are more dynamic than in those with low expression (**Figure** **5**e). We further explored the connection between the ChIP‐seq signal values and dynamic levels, revealing that POLR2A, CTCF, and DNase are enriched in the top 5% of interactions by dynamic index (Experimental Section). This enrichment is closely associated with the transcription factor binding site (Figure S13, Supporting Information). These findings underscore the pivotal role of regulatory molecules in driving chromatin dynamics.

### 2.6 Chromatin Regulators Dynamically Modify Chromatin Conformation

To obtain the chromatin regulators that affect chromatin conformation at corresponding locations, we used the IntegratedGradients (IG) algorithm^[^ ^26^ ^]^ to calculate the association score for each feature related to the specified interaction. By aggregating the results from all interactions, we obtained the collective association score for chromatin regulators (Experimental section; Table S3, Supporting Information). We further evaluated the top 100 chromatin regulators, which highlight the importance of these factors and their score for precise prediction (Figure S14, Supporting Information).

SCENIC^[^ ^27^ ^]^ was used to further validate the meaningfulness of predicted chromatin regulators in regulating target genes. Based on previous analyses (Figure 2b) and related reports,^[^ ^28^, ^29^, ^30^, ^31^ ^]^ three marker genes were used for analysis in each cell subtype (Astro: *SLC1A2*, *SLC1A3*, and *GPM6A*; OPC: *PDGFRA*, *PTPRZ1*, and *VCAN*; ODC: *MBP*, *QKI*, and *ENPP2*). The results showed that the critical TF genes predicted by SEE were strongly correlated with the predictions of SCENIC (Figure S15a, Supporting Information). The significant overlap (≈65%) between the top 10% TF genes from SEE and SCENIC reinforces similar conclusions (Figure S15b, Supporting Information).

Next, we trained separate models for the promoter, body, and terminator positions of the gene and calculated the respective chromatin regulators (Methods). The enrichment heatmap and 3D structure revealed significant differences in gene regulation patterns across cell subtypes or genes (Figure S16, Supporting Information). Specifically, for the *MBP* gene under the ODC cell subtype, we observed a high similarity between the promoter and the terminator positions. Furthermore, it was apparent in the 3D structure diagram that the promoter and terminator were situated close (Figure S16c,d, Supporting Information). These results suggested that chromatin regulators dynamically modify the chromatin conformation of the *MBP* gene, leading to the proximity of the promoter and terminator positions.^[^ ^32^ ^]^

Enrichment patterns of transcription factors across different cell subtypes highlighted the heterogeneity in gene expression, which aligns with the trajectory of cell differentiation. *PDGFRA* changed from open to closed during the differentiation process from OPC to ODC.^[^ ^33^ ^]^ As a transcriptional repressor,^[^ ^34^ ^]^ *ZBTB20* showed strong reverse enrichment at the promoter within OPC; *ST18*, a marker gene for ODC, was only enriched in ODC (Figure S16b, Supporting Information). As OPC differentiated into ODC, *MBP* turns from off to on. *ZBTB20* was depleted at the promoter position; *CNP*, which contributes to the formation of myelin in ODC, was exclusively enriched in ODC (Figure S16d, Supporting Information). Moreover, analysis of marker genes from different cell subtypes revealed that the regulatory patterns of marker genes within the same cell subtype were more similar (Figure S17, Supporting Information).

### 2.7 Gene‐Expression‐Coupled Dynamics of Chromatin Conformation

Through the analysis of 15 differentially expressed genes (DEGs) in the ODC cell subtype, we found a positive correlation between the amplitude of chromatin volume oscillations and the amplitude of expression level oscillations (Figure 5a,b; Δmax Spearman's *ρ*  =  0.50, *P*  =  0.058; Δsum Spearman's *ρ*  =  0.38, *P*  =  0.164). Analyzing DEGs of three cell subtypes (OPC: *PDGFRA*, ODC: *PIP4K2A*, and Astro: *SLC1A2*) revealed that their characterized cell subtype had a wider oscillatory distribution in volume than other cell subtypes (Figure S12c, Supporting Information). We further analyzed the variance similarity of the distribution of gene Δvolume under different cell subtypes and observed that for genes with high variance similarity, the overall change amplitude of gene expression was significantly similar (Figure 5c,d).

According to these findings, we propose that chromatin structure is a flexible foundation for regulatory mechanisms. Unexpressed genes, such as PDGFRA_Astro, PDGFRA_ODC, and SLC1A2_ODC (Figure 5c,d; Figure S12b, Supporting Information), usually exhibit stably low oscillations (Figure 5c; Figure S12c, Supporting Information). Such genomic regions, likely due to the lack of critical factors from the ‘village’,^[^ ^35^ ^]^ tend to maintain a stable state in regions relevant to their expression. For genes with significant change or high expression levels, such as PIP4K2A_ODC, PIP4K2A_OPC, and SLC1A2_Astro (Figure 5c,d; Figure S12b, Supporting Information), frequent and flexible transcription is often required in cells (Figure 5c; Figure S12a, Supporting Information). Changes in their expression may be regulated by internal and external signals, including factors such as transcription factors and hormones (Figure S16, Supporting Information). Constant changes in these signals will cause unstable oscillations in chromatin structure, resulting in temporal and spatial fluctuations in gene expression (Figure S12a,b, Supporting Information). Consistent with our previous study,^[^ ^36^ ^]^ this conformational change will facilitate the regulation of gene accessibility, allowing regulatory molecules to interact more easily with regulatory regions of genes (Figure 5e; Figure S13, Supporting Information).

Taken together, these results demonstrate that chromatin regulators act on the genomic loci, inducing oscillations in multiple interactions, which result in the above‐mentioned changes at the gene and TAD scales.

### 2.8 Interpreting Disease‐Associated SNPs with Dynamic Index

Using scanorama^[^ ^37^ ^]^ to integrate RNA expression data from different experiments into the same space, SEE can predict the Hi‐C maps associated with untrained RNA expression data through label transfer (**Figure** **6**a). Evaluation on bulk data indicated that SEE's predictive performance is enhanced when the untrained cell subtype resembles trained cell subtypes (Figure S18, Supporting Information).

Alzheimer's disease (AD) is a common chronic neurodegenerative disease. Although several common and rare gene expressions have been identified, the interpretation of these gene expressions in terms of chromatin conformation remains scarce. Therefore, we examined whether chromatin interaction dynamics is associated with driving disease‐specific biology through the evolution of gene regulatory programs.

Dynamic chromatin interactions play a role in interpreting the gene expression regulation mediated by genome‐wide association study (GWAS) loci. Grubman et al.^[^ ^38^ ^]^ reported that the *TFEB* gene, specifically upregulated in AD astrocytes, acts upstream of 10 GWAS loci, resulting in dysregulation of gene expression (Figure S19, Supporting Information). We selected the most remarkable examples of increased expression (*BIN1*) for further investigation. First, applying SEE to the Morabito et al. dataset^[^ ^39^ ^]^ found that chromatin interactions featuring dynamic properties exhibit greater numerical changes under diseased conditions (Figure 6b). This result revealed that changes in biological events under diseased conditions drive changes in chromatin interactions (Figure S13, Supporting Information). Second, we collected candidate AD SNPs including SNPs in high linkage disequilibrium (LD) (r2>0.8 in 1000 Genome EUR) with the reported lead SNPs of AD. Our results revealed that rs10207628 exhibits a dynamic chromatin interaction (dynamic index within top 5%) with the *cis*‐regulatory element in the Astro cell subtype (Figure 6c), suggesting that rs10207628 is likely a GWAS locus targeted by *TFEB*.

In the analysis of DEGs, SEE enables us to further decipher the mechanisms behind key genes identified to be colocalized with AD GWAS loci. As an AD risk gene, *KAT8* is differentially expressed in the Astro cell subtype. The expression Quantitative Trait Loci (eQTL) of *KAT8* was colocalized with AD GWAS loci in the prefrontal cortex (posterior probability > 0.9 by HYPRCOLOC^[^ ^40^ ^]^) with rs11865499 as the colocalized variant (Figure S20a, Supporting Information). Subsequently, SEE was employed to scrutinize the connection between these loci and *KAT8*, revealing that rs55766044 in high LD (>0.9) with rs11865499 exhibited a dynamic chromatin interaction with the promoter region of *KAT8* in Astro cell subtype (Figure S20b, Supporting Information). This interaction is upregulated in the disease state, thereby strengthening the regulatory impact on *KAT8*.

## 3 Discussion

In this study, we developed SEE, a single‐cell method to learn the relationship between gene expression and chromatin conformation. The input of SEE is scRNA‐seq data for all genes, and the output is scHi‐C information for a single specific region. For an efficient balance between computational load and accuracy, we recommend using SEE at the megabase scale or smaller.

We found that the predicted scHi‐C maps exhibit clustering patterns based on variations in cell subtypes and states. This observation underscores SEE's ability to capture subtle differences in scRNA expression data, which are subsequently reflected in scHi‐C maps. Moreover, SEE enables comparative chromatin analyses from TAD to interaction, from discrete cell groups to continuous processes, from gene to regulation, and from discovery to investigation. Importantly, by joint analysis of dynamic index and GWAS regulation system, SEE uncovered biological insights that were not captured by current single‐cell analyses based on mean changes and/or cell clusters.

Chromatin dynamics were typically investigated using imaging technologies like seqFISH+,^[^ ^41^, ^42^ ^]^ focusing on specific loci. With technological advancements, smFRET proposed by Willhoft et al. has been utilized to observe the structural dynamics of nucleosomes in real time.^[^ ^43^ ^]^ Additionally, Di Stefano et al. modeled chromatin dynamics using time‐series Hi‐C data, but were limited by population averages.^[^ ^44^ ^]^ Recently, a series of experimental techniques has emerged, enabling simultaneous capture of scRNA‐seq and scHi‐C data. This progress allowed single‐cell level investigation of whole genome chromatin dynamics, but was constrained by sensitivity and throughput^[^ ^5^, ^6^, ^7^, ^8^ ^]^ (Table S2, Supporting Information). Utilizing scRNA‐seq and limited scHi‐C data, SEE explores chromatin dynamics across different scales, and elucidates pathogenic processes by analyzing chromatin dynamics.

Due to the reliance on single‐cell omics data, the current version of SEE relies on the concept of pseudo‐time to indirectly distinguish cell identity and cell stage. The concept of pseudo‐time requires a task‐oriented definition to accurately describe dynamic features. To date, image‐based methods are available to examine spatiotemporal gene expression at endogenous loci within the context of living cells. It is reasonable to expect that image‐based methods can reveal further insights into the cell stage. Furthermore, it is necessary to integrate more types of omics data to capture additional information for explaining chromatin dynamics, including protein, non‐coding RNA, and histone modification.^[^ ^45^, ^46^, ^47^ ^]^ Chromatin dynamics studies will gradually unravel the mechanisms of gene expression regulation, such as transcription timing and transcription patterns.^[^ ^48^ ^]^ Understanding the principles of chromatin dynamics is essential for gaining insight into development and disease processes.

Overall, SEE facilitates the analysis of chromatin dynamics by integrating unpaired single‐cell chromatin conformation data and transcriptome data. Through the large amount of information generated computationally, SEE can observe chromatin dynamics and its intricate interplay with transcription across various temporal and spatial scales. The entire SEE code and associated analyses are available online (https://github.com/4dglab/SEE).

## 4 Experimental Section

### Overview of SEE

SEE considers a scRNA expression dataset of *N_R_* cells $D_{R}=\{{g^{i}\}}_{i=1}^{N_{R}}$ and a scHi‐C maps dataset of *N_H_* cells $D_{H}=\{{c^{j}\}}_{j=1}^{N_{H}}$, where $g^{i}\in R^{K_{R}}$ and $c^{j}\in R^{K_{H}\times K_{H}}$ are the gene expression vector of cell *i* with *K_R_* genes and the contact matrix of cell *j* with *K_H_* bins. The goal of SEE is to learn a function $f_{SEE}\;:g\to \hat{c}$ that maps the novel gene expression vector **g** to local chromatin conformation $\hat{c}$, which is the contact matrix **c** with *K_h_* < *K_H_*.

### Construction of a Mapping from scRNA Samples to scHi‐C Samples

The mapping of scRNA and scHi‐C data and the production process of training data are shown in Figure S1 (Supporting Information).

*scRNA and scHi‐C Data Preprocess*: The scRNA data for this work are from the Allen Cell Types Database, which covers multiple cell subtypes. The cell subtypes were filtered based on whether the sample size ratio of cell subtypes in scHi‐C data and scRNA data is similar. The scRNA expression dataset $D_{R}$ is finally processed into a cell‐by‐gene matrix, $X_{R}\in R^{N_{R}\times K_{R}}$, where each element $X_{R}^{i,j}\in R^{+}$ represents the expression count of RNA molecule *j* on cell *i*. The scHi‐C maps for this work are from Lee2019 (human) and Tan2021 (mice). The source data are .cool and .hic format files, respectively, and we stipulate that every 10‐Kb is used as a bin to obtain the contact matrix **c**. Then, $D_{H}$ is processed into a cell‐by‐bin matrix, $X_{H}\in R^{N_{H}\times K_{H}}$, where each element $X_{H}^{i,j}\in R^{+}$ represents the sum of contact values with other bins of 10‐Kb bin *j* on cell *i*. The preprocessed scRNA expression data and scHi‐C maps are stored in the .h5ad format to reduce the computational and time costs in subsequent processing.

*Dimensionality Reduction for scRNA Expression Data and scHi‐C Maps*: The scRNA expression data $D_{R}$ is subjected to analysis workflow in Scanpy as follows. First, select genes whose sum of expression counts is greater than 0. Then, the highly variable genes (2000 genes) of the scRNA data are calculated with seurat_v3 as the flavor. Next, the filtered data are handled by the normalize_total function and then log1p‐transformed and scaled. Finally, the scRNA data are dimensionally reduced using the PCA method (100 components, auto svd_solver). The scHi‐C maps $D_{H}$ are dimensionally reduced using the BandNorm^[^ ^49^ ^]^ under default parameters. The dimensionality reduction result of scRNA expression data and scHi‐C maps are denoted as $X_{R}\in R^{N_{R}\times K_{D}}$ and $X_{H}\in R^{N_{H}\times K_{D}}$, where *K_D_* ≡ 100 in this work. The RNA and Hi‐C clustering is performed using the first 100 principal components of PCA $X_{R}$ and BandNorm $X_{H}$ with Scanpy “pp.neighbors(metric = ‘cosine’)” and “tl.leiden” functions.

*Association Graph*: Considering the co‐localization of 1D, 2D, and 3D information between scRNA data and scHi‐C data on chromatin fragments, an association graph G=(V,E) was build to represent the relationship between features of them. $V=\{{G^{i}\}}_{i=1}^{K_{R}}\;\cup \{{B^{j}\}}_{j=1}^{K_{H}}$ is the universal feature set, where *G^i^* and *B^j^* are gene name in scRNA expression data (Column *i* in *X_R_*) and bin in scHi‐C maps (Column *j* in *X_H_*). E={(i,j)|i,j∈V} is the set of edges. Each edge contains distance *d*_*i*,*j*_ and weight *w*_*i*,*j*_. Finally, the edges between two different omics features were defined as:

(1) $$d\;\left(G^{i},B^{j}\right)=\left\{\begin{array}{c} abs\left(s_{G}^{i}-s_{B}^{j}\right)\times f_{\mathrm{map}}\left(u_{G}^{i},u_{B}^{j}\right),\;\;h_{G}^{i}=h_{B}^{j}\\ inf,\;\;\;\;\;\;\;\;\;otherwise \end{array}\right.$$

(2) $$w\;\left(G^{i},B^{j}\right)={\left(\frac{d\left(G^{i},B^{j}\right)+10000}{10000}\right)}^{-0.75}$$

where

(3) $$f_{\mathrm{map}}\;\left(u_{G}^{i},u_{B}^{j}\right)=\left\{\begin{array}{c} 1,\;\;\;\;\;\left[s_{G}^{i},e_{G}^{i}\right]\cap \left[s_{B}^{j},e_{B}^{j}\right]=\emptyset \\ 0,\;\;\;\;\;\;\;\;\;otherwise \end{array}\right.$$

***u*** represents the position information (chromatin ***h***, start position ***s***, end position ***e***) of each feature (gene names ***G*** and bins ***B***). *i* represents the number of features. [*,*] represents the interval range.

Due to the large number of features in scRNA expression data and scHi‐C maps, the time complexity of traversing the relationships between each feature is too high. The window_graph function (Algorithm 1) was defined, which enables the method to quickly and effectively examine the correlation between the features of different omics, so as to generate a more reasonable association graph.

Algorithm 1 **Association graph construction**

**Table:** 

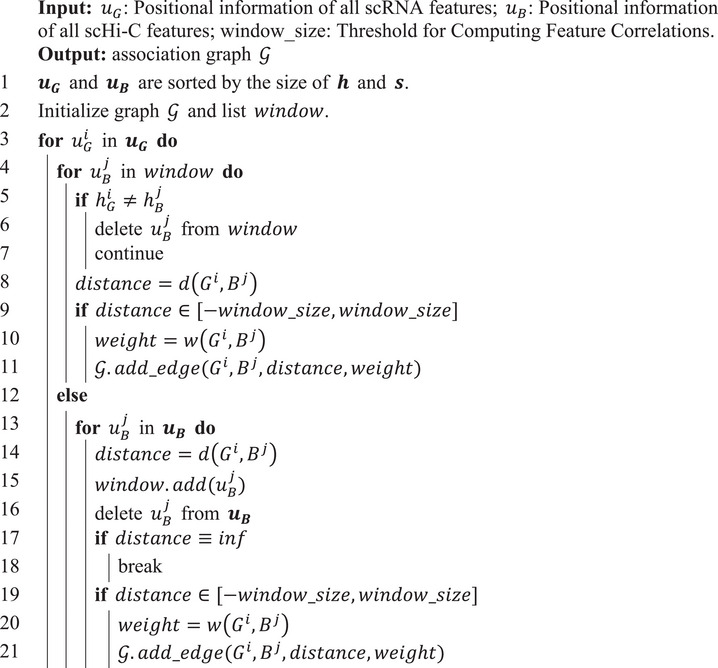

*Integration of scRNA‐Seq Data and scHi‐C Data*: The dimensionality reduction result of scRNA expression data $X_{R}$ and scHi‐C maps $X_{H}$ are used as the correlation relationship between each feature in each omics data; the association graph of scRNA expression data and scHi‐C maps G solves the problem of semantic inconsistency during dimensionality reduction. Through low‐dimensional information and association graph, data aggregation algorithms can effectively guide the mutual supervision between/among the two omics data features. In this work, we use GLUE implemented in scglue/v. 0.3.2 as the data aggregation algorithm.^[^ ^50^ ^]^ Specifically, the association graph was used as the prior guidance graph in scglue, and propagate highly variable scRNA features (genes) to identify highly variable scHi‐C features (bins) based on the association graph. We then build and train the GLUE integration model using the PCA and BandNorm embeddings ($X_{R}$ and $X_{H}$) as the first encoding transformation, using the raw counts of scRNA expression data and scHi‐C maps (*X_R_* and *X_H_*) and the prior guidance graph G as input. After the GLUE model is trained, integrated cell embeddings were calculated, $\hat{X}\in R^{\left(N_{R}+N_{H}\right)\times K_{D}}$, for both scRNA and scHi‐C data using scglue. Finally, UMAP dimensionality reduction was performed and visualized it (Figure 1b right; Figures S2a and S8a, Supporting Information) using the same procedures as previously described.

*Construction of scRNA‐to‐scHi‐C Dataset using the Same Latent Space Information*: After using cell embeddings $\hat{X}$ to calculate neighbors with Scanpy, we can get the connectivity values (connectivities) of all samples (including scRNA and scHi‐C) in the low‐dimensional space. SEE uses connectivities as the primary mapping metric. It is stipulated that, under the premise of the same cell subtype, each scRNA sample data is paired with the scHi‐C sample data with the highest connectivity value. The scRNA‐to‐scHi‐C relationship is defined as:

(4) $$L\;=\left\{{\left.\left.\left(I_{R}^{i},I_{H}^{f_{\mathrm{mapping}}\left(I_{R}^{i},I_{H}\right)}\right)\right|f_{\mathrm{maxcon}}\left(I_{R}^{i},I_{H}\right)\neq -1\right\}}_{i=1}^{N_{R}}\right.$$

(5) $$f_{mapping}\;\left(I_{R}^{i},I_{H}\right)=argmax_{j=1,\text{…},N_{H}}\;f_{con}\left(I_{R}^{i},I_{H}^{j}\right)$$

(6) $$f_{maxcon}\;\left(I_{R}^{i},I_{H}\right)=max_{j=1,\text{…},N_{H}}f_{con}\left(I_{R}^{i},I_{H}^{j}\right)$$

(7) $$f_{con}\;\left(I_{R}^{i},I_{H}^{j}\right)=\left\{\begin{array}{c} connectivities\left(I_{R}^{i},I_{H}^{j}\right),\;same\;cell\;type\\ -1,\;\;\;\;\;\;\;\;otherwise \end{array}\right.\;$$

where **I**_*R*_ and **I**_*H*_ represent scRNA and scHi‐C sample indices. *i* and *j* represent the numbers of scRNA and scHi‐C samples. $L={\left\{\left(I_{R}^{i},{I^{\prime }}_{H}^{i}\right)\right\}}_{i=1}^{N}$ represents the link from scRNA samples to scHi‐C samples.

Next, the one‐to‐one correspondence was denoted between the input and output of the model by $D={\left\{\left(g^{i},c^{i}\right)\right\}}_{i=1}^{N}$, where **g** ^*i*^ and **c** ^*i*^ are gene expression vector and local scHi‐C map of sample *i* in L, which is consistent with the previously description (section Overview of SEE). For efficient inference and optimization, evaluations generally focus on predicting local scHi‐C map of marker gene fragments. The screening of target gene fragments is as follows:

First, the same analysis process was used as the “Dimensionality reduction for scRNA expression data and scHi‐C maps” step to reduce the dimensionality of the scRNA data. Then, the genes that characterize the population are ranked (using the t‐test method) by Scanpy::rank_genes_groups to determine the marker gene for each cell subtype. Since the data resolution is 10‐Kb, genes that are too short cannot play a suitable training role, so we further screen the predicted target genes with 50‐Kb as the minimum threshold to obtain a list of trainable genes.

### Model Architecture

The architecture of the SEE model is shown in Figure 1b down. First, one cell's RNA expression vector **g** ^*i*^ as input data is taken, which is then converted to (8 × *m*, 8 × *n*) format by reshape function. Next, the Encoder encodes the transformed data to obtain a set of latent vectors. Finally, this latent vector is input into the Decoder to infer the upper triangular data of local scHi‐C map $\hat{o}$.

For the step of reshaping the RNA data into (8 × *m*, 8 × *n*), since the data information density is very low, the input data was directly cut so that the data can be divisible by 64 (the number of cut dimensions is much smaller than all dimensions quantity). Then, *K_R_*/64 was factorized to get *m* and *n* (both *m* and *n* are integers, and *m* and *n* need to be as close as possible in value).

The Encoder and Decoder parts of the model use a transformer network instead of a CNN network for the following two reasons. First, each feature of the input data has indefinite long‐distance dependence rather than spatial locality. Second, the total amount of input data information is large (the feature dimension is greater than 10 000), but the information density is low (the feature pattern is less than ten).

The Encoder uses the Encoder part of the Vision Transformer (ViT) because of its strong global space modeling ability and its capability to learn feature dependencies. Compared with ordinary transformers, ViT integrates multiple features into one patch, which effectively reduces the complexity of the model. Specifically, the number of receiving channels of Encoder is set to 1, patch_size is set to *m* × *n*, num_classes is set to 1000, dim is set to 1024, depth and heads are set to 6 and 8, and mlp_dim is set to 2048. In this study, Encoder first divides the input data into 64 patches by patch_size and uses Linear Projection to embed each patch. Next, each patch embedding and the position embedding of the corresponding position are put into the backbone network of Encoder to obtain a latent vector.

The Decoder consists of two fully connected layers and a transformer's Encoder. For convenience, this Encoder was called as a Decoder. The first fully connected layer was responsible for mapping the potential vector output by the Encoder to the input dimension of the Decoder. The second fully connected layer is responsible for converting the vector output by the Decoder into the corresponding scHi‐C upper triangular map information. Specifically, the Decoder's dim is set to 512, depth and heads are set to 6 and 8, and mlp_dim is set to 2048.

### Loss Function and Training Details

Training dataset exhibits a significant imbalance in its data distribution. In order to make the model pay more attention to the data with disproportionately large loss values, the known focal loss algorithm was modified and used it as the loss of model training:

(8) $$L\;\left(\hat{o},o\right)=\frac{1}{N_{b}}\;\sum_{i=1}^{N_{b}}\left({\left(1-e^{-L_{MSE}\left({\hat{o}}^{i},o^{i}\right)}\right)}^{\gamma }\right)\times L_{MSE}\left({\hat{o}}^{i},o^{i}\right)$$

where

(9) $$L_{MSE}\;\left({\hat{o}}^{i},o^{i}\right)=\sum_{j=1}^{N_{c}}{\left({\hat{o}}^{i,j}-o^{i,j}\right)}^{2}$$

$\hat{o}$ and $\in R^{N_{b}\times N_{c}}$, represent the upper triangular matrix vectors of predicted**/**source scHi‐C map in one batch. *i* ∈ [1, *N_b_*] is the index of batch; *j* ∈ [1, *N_c_*] is the index of interaction. **o** ^*k*^ can be obtained through Algorithm 2 and local scHi‐C map **c** ^*k*^. γ is a hyperparameter defined by focal loss. In this study, it is set to 2 for training.

Algorithm 2 **Extracting upper triangular matrix vector**

**Table:** 

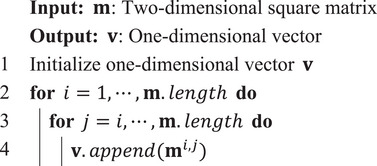

In terms of computational resources, the SEE model was trained on a single‐core 3090 GPU with 24GB of memory. With 58 million parameters, the SEE model utilizes ≈ 7.57 GFLOPs for a single forward pass. A learning rate of 0.0001 was used to train the model. On the Lee2019 dataset,^[^ ^10^ ^]^ the batch_size was set to 8. On the Tan2021 dataset,^[^ ^19^ ^]^ the batch_size was set to 4. During training with 8 batch_size, the model consumes ≈3000 MB of GPU memory, which includes space for gradients, feature maps, and other training artifacts. All codes were completed on the Linux operating system. It is recommended to use devices with 128GB memory and storage larger than 1TB for end‐to‐end experiments to avoid encountering OOM‐like errors. The codes related to the environment, training, prediction, and analysis are all publicly available in the GitHub. The entire framework of SEE was divided into preprocessing, integration, training, and analysis. Only the training stage requires users to specify the chromatin region, using one parameter.

### The Dynamics of Chromatin Conformation

*Trajectory Analysis of scRNA‐Seq Data*: The coarse‐grained connectivity structures of complex manifolds were generated using the first 100 principal components of PCA $X_{R}$ with Scanpy “pp.neighbors(metric = `cosine`)”, “tl.umap”, “tl.leiden”, and “tl.paga” functions. Then, we infer the progression of cells (pseudo‐time $T=\{{t^{i}\}}_{i=1}^{N_{R}}$) with Scanpy::tl.dpt and perform force‐directed graph with the initial position from PAGA (Scanpy::tl.draw_graph) to visualize the development trajectories (Figure 3a; Figure S10a, Supporting Information).

*Pseudotemporal Dynamics of Gene Expression and Chromatin Conformation*: In the first step of the process, the pseudo time‐order scRNA expression data was obtained and predicted scHi‐C maps. The trained model was applied to perform the scHi‐C prediction:

(10) $$\;\hat{D}={\left\{\left(g^{i},{\hat{c}}^{i}\right)\right\}}_{i=1}^{N_{R}}$$

(11) $$\;{\hat{c}}^{i}=f_{SEE}\left(g^{i}\right)$$

A permutation δ was defined that satisfies a bijection from the set {1, 2, ⋅⋅⋅, *N_R_*} to itself:

(12) $$\forall i,j\in \left\{1,2,\text{…},N_{R}\right\},i<j\Rightarrow T\left[\delta \left(i\right)\right]\leq T\left[\delta \left(j\right)\right]$$

Then, the data $\hat{D}$ was sorted in ascending order according to δ:

(13) $$\;\hat{D_{T}}={\left\{\left(g^{\delta \left(i\right)},{\hat{c}}^{\delta \left(i\right)}\right)\right\}}_{i=1}^{N_{R}}$$

In the second step of the process, the changes in features over pseudo‐time are quantified. For the diagrams in Figure 3b and Figure S10b (Supporting Information), the expression count of *MBP* and *PDGFRA* was visualized. In order to more intuitively represent changes in chromatin conformation, we first randomly sample the interactions on the predicted scHi‐C map based on their contact values, defined as $P=\{\left(p^{\delta (1)},t^{\delta (1)}\right),\text{…},\left(p^{\delta (N_{R})},t^{\delta (N_{R})}\right)\}$, where *p* ^δ(*k*)^ = (i^δ(*k*)^,j^δ(*k*)^)  is the position of the interaction sampled in the *k*‐th sample of $\hat{D_{T}}$. The position of each interaction was further randomized within a 10‐Kb range of the original position in P, defined as 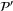, which is also used for the diagrams in Figures S10c and S11b (Supporting Information). Then, $M\left(n\right)\in R^{K_{h}\left(n\right)\times K_{h}\left(n\right)}$ was denoted as super‐resolution matrix to describe the pseudotime‐order migration of primary interaction, where $\exists n\in Z^{+}\Rightarrow K_{h}\;\left(n\right)=n\times K_{h}$. In this work, M(2) was populated with information from 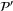:

(14) $$\forall a,b\in \left\{1,2,\text{…},2\times N_{R}\right\}\Rightarrow \;\;M{\left(2\right)}^{a,b}=\frac{1}{N}\;\sum_{\left(p,t\right)\in P^{\prime }:i\in \left[\frac{a}{2},\frac{a+1}{2}\right]\wedge j\in \left[\frac{b}{2},\frac{b+1}{2}\right]}t$$

and convert it into a continuous matrix M(10) with Scipy::interpolate.griddata with cubic interpolation (Figure 3c; Figure S11a, Supporting Information).

*Chromatin Volume*: The chromatin structure was reconstructed from Hi‐C map using 3DMax^[^ ^25^ ^]^ with parameters (*NUM*  =  1, *VERBOSE*  =  *true*, LEARNING_RATE=1 and MAX_ITERATION=10000). Then, 3D coordinates of all loci, derived from 3DMax, are used to calculate a convex hull with Scipy::spatial.ConvexHull. The volume of this convex hull was defined as the chromatin volume.

*Interaction Dynamic Score*: The vibration score and switch score are calculated by analyzing the differences between Hi‐C maps over pseudo‐time. The vibration score represents the average of the absolute value of ΔscHi‐C. The dynamic index is obtained by sorting the vibration scores of a large number of interactions. Moreover, each ΔscHi‐C matrix is compared with the vibration score to generate a binary matrix with the absolute value of the element greater than the vibration score being 1, otherwise 0. All the binary matrixes are averaged to obtain the switch score map. Specifically, let $D_{\delta }={\left\{\delta_{c}^{i}\right\}}_{i=1}^{N_{R}-1}$ represent the collection of differences between adjacent Hi‐C map in pseudo‐time ordering, where $\delta_{c}^{i}=c^{i+1}\;-c^{i}$ is ΔscHi‐C in Figure 4d. Afterward, $X_{v}\in R^{K_{h}\times K_{h}}$ was denoted as vibration score map, which satisfies:

(15) $$\forall a,b\in \left\{1,2,\text{…},K_{h}\right\}\Rightarrow \;X_{v}^{a,b}=\frac{1}{N_{R}-1}\;\sum_{i=1}^{N_{R}-1}abs\left(\delta_{c}^{i}\left(a,b\right)\right)$$

Again, $X_{s}\in R^{K_{h}\times K_{h}}$ was defined as switch score map, which satisfies:

(16) $$\forall a,b\in \left\{1,2,\text{…},K_{h}\right\}\Rightarrow \;X_{s}^{a,b}=\frac{1}{N_{R}-1}\;\sum_{i=1}^{N_{R}-1}\left[abs\left(\delta_{c}^{i}\left(a,b\right)\right)>X_{v}^{a,b}\right]$$

### The Importance of Chromatin Regulators

*Auxiliary Data*: To effectively analyze which genes affect the changes in chromatin conformation, we use the human‐related TF list and TF Cofactors list recorded in the AnimalTFDB 3.0^[^ ^51^ ^]^ databases and the human‐related RBP list recorded in the EuRBPDB^[^ ^52^ ^]^ database.

*Definition and Setting of Baseline*: Human beings usually rely on counterfactual intuition when making attributions. When humans attribute certain responsibilities to a certain cause, they implicitly use the absence of the cause as the baseline for comparison. Based on the principle of human attribution, deep network attribution also needs a baseline to simulate the absence of reasons. In this study, it is believed that the expression of genes will affect the 3D structure of our own/other genes. When all genes are not expressed, the 3D structure of chromatins should be stable, so it is decided to use all eigenvalues ​​set to 0 as the baseline.

*Feature Association Score*: Integrated gradients represent the gradient integral with respect to the input on the process from a given baseline to a real input. For specific cases, the algorithm is described in this study as:

(17)

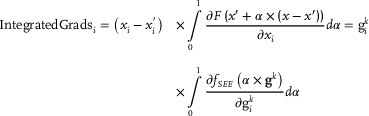

where $\frac{\partial F(x)}{\partial x_{i}}$ is the gradient of *F*(*x*) in the *i* dimension, and **g** ^*k*^ is a sample in **g**. Since the output of *f_SEE_* is a 2D matrix $\hat{o}$, IntegratedGrads was redefined as:

(18) $${\;\mathrm{IntegratedGrads}}_{i}^{j}=g_{i}^{k}\;\times \int_{0}^{1}\frac{\partial F_{j}\left(\alpha \times g^{k}\right)}{\partial g_{i}^{k}}d\alpha$$

where *F_j_* (*x*) is the *j*‐th feature of *f_SEE_*.

*The Association Score of Chromatin Regulators to the Target 3D Structure*: The input data (scRNA) of the model has multiple modes (Astro, OPC, etc.), and different data modes will affect the weight assignment of the model to features. Therefore, when analyzing model feature association, the input data is divided by cell type. For example, *PDGFRA* is a marker gene of OPC. When calculating the feature association of its related models, only data related to OPC cell subtypes are used. We define the feature association calculated by the *PDGFRA* correlation model after using OPC data as the gene correlation coefficient of *PDGFRA*. Features with higher scores (chromatin regulators) are more likely to affect the chromatin conformation of *PDGFRA*.

Since the IG algorithm in the captum toolkit can only attribute one value of the target result at a time, Algorithm 3 was proposed to quantify the association score of chromatin regulators:

Algorithm 3 **Aggregating the contributions from all dimension**

**Table:** 

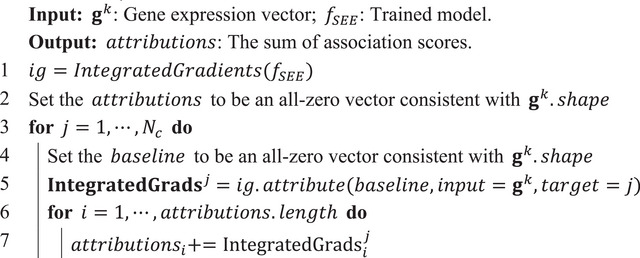

### Effectiveness Testing

SEE was validated in 34 randomly selected genomic regions across the whole genome, including 10 regions with marker gene, 12 regions with housekeeping gene, and 12 domain regions. The marker gene was identified based on prior computational results (Figure 2b), with *AQP4* excluded due to its short genomic length. For the regions with housekeeping gene, 12 genes with lengths of at least 200 kb were randomly chosen from the Housekeeping Transcript Atlas (https://housekeeping.unicamp.br/Housekeeping_GenesHuman.csv), with the gene bodies serving as the training regions. For the domain regions, the selection process involved generating target pseudo‐bulk Hi‐C map, calculating insulation scores to identify candidate boundaries, and then randomly selecting 12 boundaries. The corresponding training regions were defined as the genomic segments spanning from each selected boundary to the next.

The prediction performance for each cell subtype was evaluated separately. The Pearson correlation coefficients were calculated between the predicted pseudo‐bulk Hi‐C maps and the target pseudo‐bulk Hi‐C maps (Table S1, Supporting Information). The results were also visualized and made available online (https://scce.readthedocs.io/en/latest/genomewide_evaluations.html).

### Evaluation of Chromatin Regulators Identification

*Calculation of Related TF Genes using SCENIC*: For human scRNA expression data, the SCENIC was used to analyze the important TF genes of three cell subtypes (Astro, OPC, and ODC). Specifically, the TF‐gene module of SCENIC was used to calculate which TF genes in different cell subtypes participate in the regulatory network of the specified gene. The specific steps are as follows:

1. The Scanpy::filter_genes function was used to filter genes (min_cells = 10) on the previously preprocessed scRNA data *X_R_*.

2. The genes of TF, TF Cofactors, and RBP lists are combined as the TF genes concerned by SCENIC; scRNA data are divided according to different cell subtypes.

3. The grnboost2 function. The input parameters are tf_names and scRNA data of specified cell subtype (obtained in the previous step). The output results of function are three columns (TF gene, target gene, importance score).

*Comparison of the Inference Result Between SEE and SCENIC*: Since the analysis of related genes for a single gene cannot fully represent the relevant cell subtypes, three related marker genes were selected for each cell subtype: Astro uses *SLC1A2*, *SLC1A3*, and *GPM6A*; OPC uses *PDGFRA*, *PTPRZ1*, and *VCAN*; ODC uses *MBP*, *QKI*, and *ENPP2*.

Assuming $attributions=\left(attributions_{1},\text{…},attributions_{K_{R}}\right)\;\in R^{K_{R}}$ as the inference result of IG, we first normalize the individual inference results by $\frac{attributions}{max(attributions)}$. Second, the TF list was traversed and found the data that satisfies both the IG inference result and SCENIC calculation result: 1) The TF column contains the genes recorded in the TF list. 2) The target column contains the marker gene.

These data are presented as a scatter plot with the SCENIC score on the x‐axis and the predicted IG value on the y‐axis (Figure S15a, Supporting Information). Meanwhile, the top 10% related TF genes were extracted by sorting their respective scores and then use a Venn diagram to present the overlap ratio (Figure S15b, Supporting Information).

*Comparison of the SEE's Inference Result Between Gene Promoter, Body, and Terminator*: The position of the gene promoter/terminator was defined as the region extending the gene_length/2 around the start/end position of the gene. For different target segments (promoter, body, terminator), the models were trained separately and used the IG method to infer and predict the association score of chromatin regulators.

As mentioned in the previous section, the individual inference results were first normalized by $\frac{attributions}{max(attributions)}$ to ensure that various inference results can be analyzed uniformly. Second, the results of the three positions were traversed and analyzed, and search for the top 20 chromatin regulators in each position. The union of the chromatin regulators of the three positions was taken as the analysis object of the heatmap.

### Measuring Clustering Accuracy Using ARI

The adjusted rand index (ARI) was extended from the rand index (RI), which computes a similarity measure between two clusterings by considering all pairs of samples and counting pairs that are assigned in the same or different clusters in the predicted and true clusterings. The raw RI score is then “adjusted for chance” into the ARI score using the following scheme:

(19) $$ARI=\frac{RI-E\left(RI\right)}{max\left(RI\right)-E\left(RI\right)}$$

In this study, ARI was used in the following analysis process. First, a marker gene on each of the three cell subtypes was selected for training and predicting, and the predicted local scHi‐C maps were processed directly by PCA. Then, the PCA results were further processed using the Leiden classification method, allowing the program to unsupervised classifies the results. Finally, the ARI coefficients of this classification result were calculated with scRNA expression data and compared them.

### Validation Using CTCF Signal and Alternative Splicing

The impute_BSS00089_CTCF data associated with Astro was obtained from WashU Epigenome Browser^[^ ^12^ ^]^ for validation. First, all the predicted scHi‐C maps are summed and averaged to obtain a pseudo‐bulk scHi‐C map. Then this matrix was averaged over the correlated interactions in bin units to get each bin's average correlated interaction value. When each bin was regarded as a point to analyze the relationship between the interaction value and the signal p‐value of CTCF, it revealed a positive correlation between them; the statistics were also directly performed at the single‐cell level, and the conclusion was still that there is a positive correlation between the interaction and CTCF. (Figure S6a, Supporting Information) Moreover, the average interaction of each bin in 12 random domains was calculated, and use the IDR thresholded peaks file (from ENCODE[ENCFF246XGW]) to obtain bins containing CTCF peaks. The results show that bins containing CTCF peaks have higher interaction values (Figure S6c, Supporting Information).

Splicing events were used to side‐stamp the value of interactions. First, SUPPA2^[^ ^13^ ^]^ was applied to predict splicing events for the gene range, setting the “input‐file” to all exons of the gene and the “format” to “ioe.” After predicting all event types (SE, SS, MX, RI, FL) and integrating the prediction results, it is specified that all the bins between the start and end positions of a splicing event were involved in the splicing event, and the splice number of the corresponding bin was +1 accordingly. Then, by performing the statistics in the same way as the CTCF analysis, it is found that the splice number and the interaction value were positively correlated at both the single‐cell level and the pseudo‐bulk Hi‐C level. (Figure S6b,d, Supporting Information)

### Code Availability

SEE and related tutorials are freely available to the public at GitHub https://github.com/4dglab/SEE. For reproducibility, the code for regenerating the Figures and Supporting Information has also been deposited in the GitHub repository.

## Conflict of Interest

The authors declare no conflict of interest.

## Supporting information



Supporting Information

Supporting Information

Supporting Information

Supporting Information

Supporting Information

## Data Availability

Publicly available datasets used were: Bakken et al.^[^ ^11^ ^]^ Human M1 – 10x Genomics (scRNA) at: https://assets.nemoarchive.org/dat‐ek5dbmu. Lee et al.^[^ ^10^ ^]^ Human PFC (scHi‐C) at https://www.ncbi.nlm.nih.gov/geo/query/acc.cgi?acc=GSE130711. Yao et al.^[^ ^20^ ^]^ Mouse Whole Cortex and Hippocampus 10x (scRNA) at: https://assets.nemoarchive.org/dat‐jb2f34y. Tan et al.^[^ ^19^ ^]^ Mouse cortex and hippocampus (scHi‐C) at: https://www.ncbi.nlm.nih.gov/geo/query/acc.cgi?acc=GSE146397. Morabito et al.^[^ ^39^ ^]^ Human Prefrontal Cortex Late‐stage AD&Control (scRNA and scATAC) at: https://www.synapse.org/#!Synapse:syn22079621/. Liu et al.^[^ ^5^ ^]^ developing mouse embryos (scHi‐C and scRNA) at: https://www.ncbi.nlm.nih.gov/geo/query/acc.cgi?acc=GSE223917. ChIP‐seq data and bulk Hi‐C from ENCODE data portal^[^ ^53^ ^]^ (https://www.encodeproject.org/) data and 4D Nucleome data portal^[^ ^54^ ^]^ (https://data.4dnucleome.org/).
