# Supplementary material for: SEE: A Method for Predicting the Dynamics of Chromatin Conformation Based on Single‐Cell Gene Expression
Source: Adv Sci (Weinh). 2025 Jan 7;12(8):2406413. doi: 10.1002/advs.202406413 (PMC11848634; doi:10.1002/advs.202406413)
Supplement: Supplementary file 1 — Supporting Information [file ADVS-12-2406413-s002.pdf]

ADVANCED SCIENCE
Open Access

Supporting Information

for *Adv. Sci.*, DOI 10.1002/advs.202406413

SEE: A Method for Predicting the Dynamics of Chromatin Conformation Based on Single-Cell Gene Expression

Minghong Li, Yurong Yang, Rucheng Wu, Haiyan Gong, Zan Yuan, Jixin Wang, Erping Long, Xiaotong Zhang and Yang Chen**

<u>**Supporting Information**</u>

SEE: a method for predicting the dynamics of chromatin conformation based on single-cell gene expression

- **Figure S1-S20.**
- **Video S1.**
- **Supplementary References.**

Figure S1

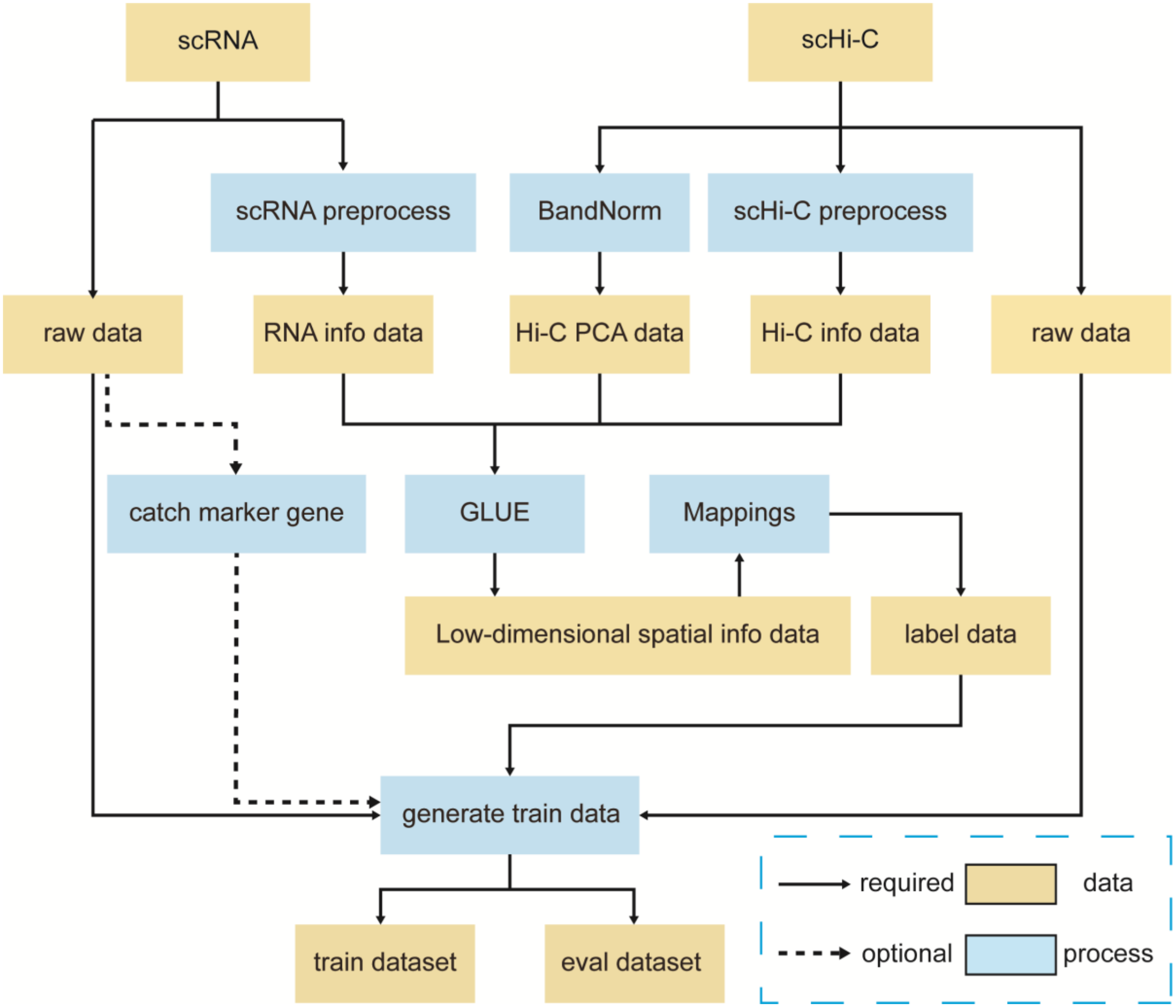

Data flow diagram about mapping scRNA and scHi-C data in SEE.

Figure S2

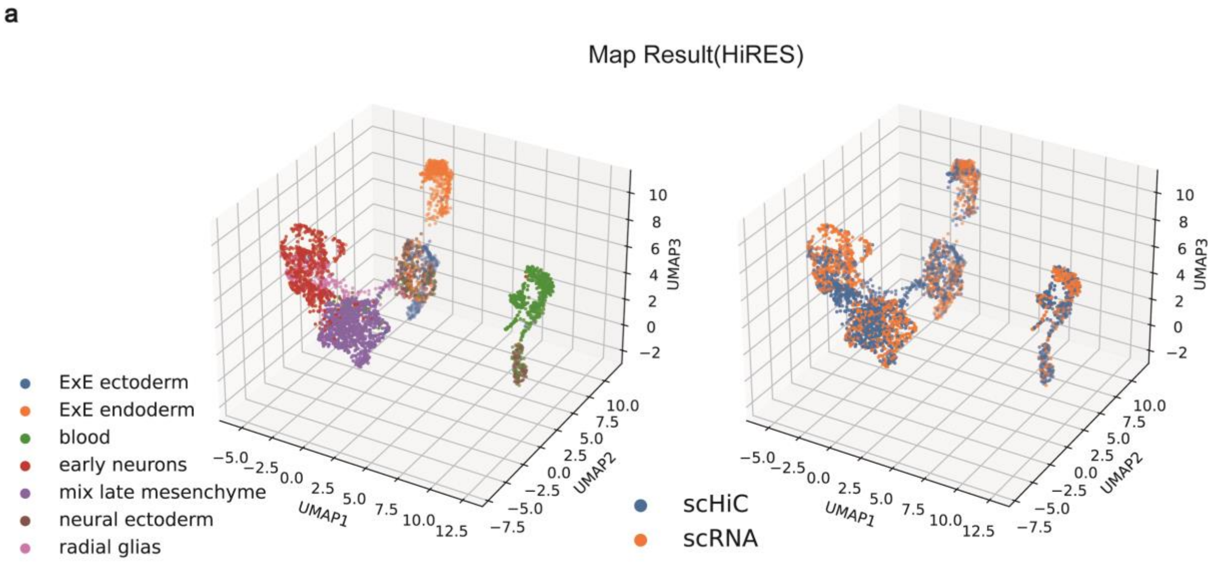

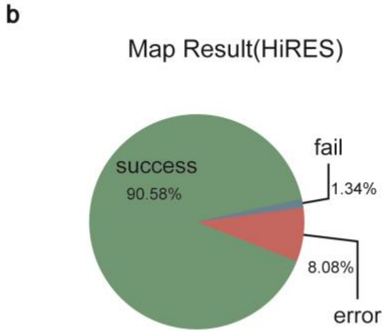

Examination of the cell embeddings in the HiRES dataset. a-b, The 3D Scatter plot (a) and Pie chart (b) of the mapping result.

Figure S3

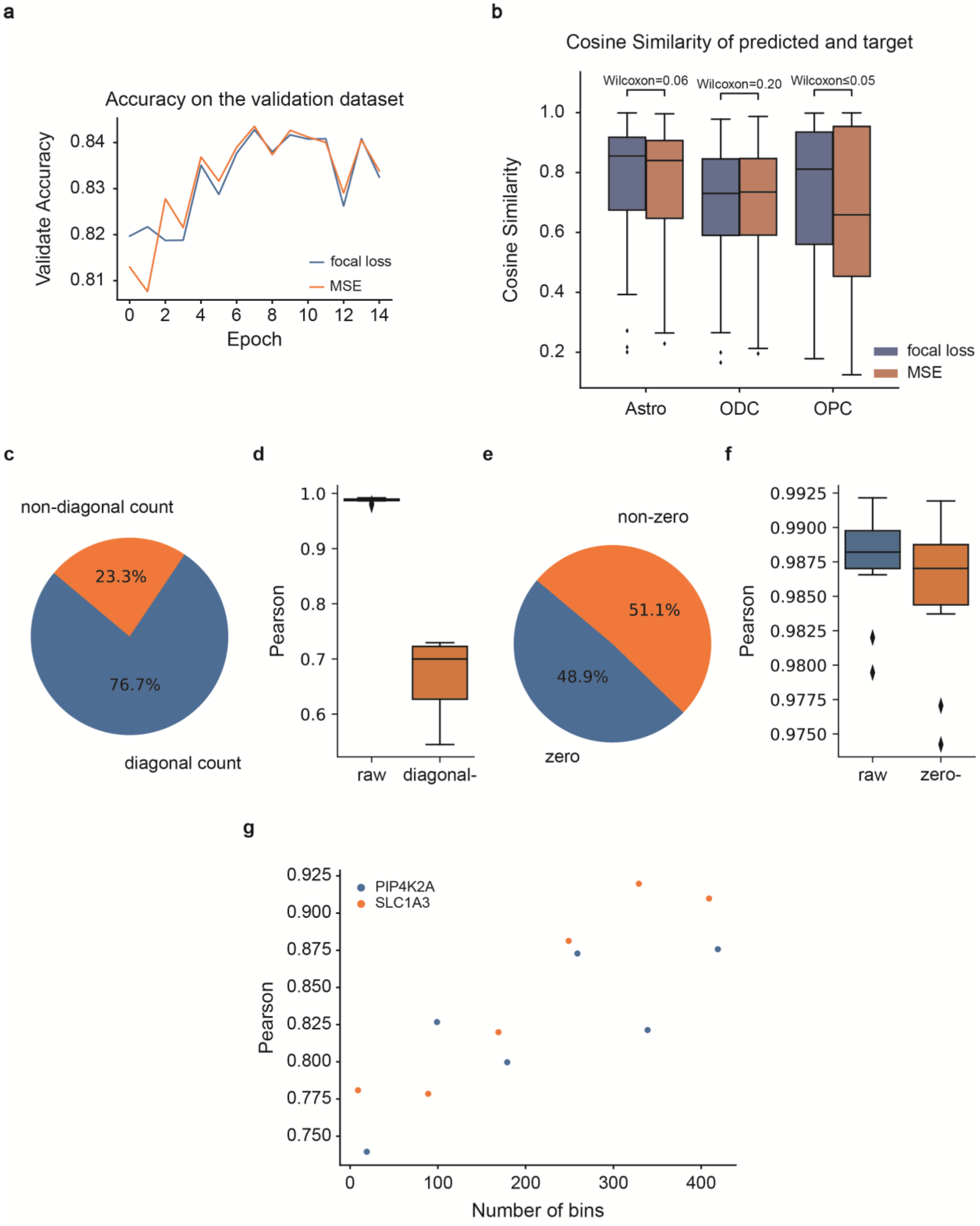
a
Accuracy on the validation dataset
Validate Accuracy
0.84
0.83
0.82
0.81
0
2
4
6
8
10
12
14
Epoch
focal loss
MSE
b
Cosine Similarity of predicted and target
Wilcoxon=0.06
Wilcoxon=0.20
Wilcoxon≤0.05
Cosine Similarity
1.0
0.8
0.6
0.4
0.2
Astro
ODC
OPC
focal loss
MSE
c
non-diagonal count
23.3%
76.7%
diagonal count
d
Pearson
1.0
0.9
0.8
0.7
0.6
raw
diagonal-
e
non-zero
51.1%
48.9%
zero
f
Pearson
0.9925
0.9900
0.9875
0.9850
0.9825
0.9800
0.9775
0.9750
raw
zero-
g
PIP4K2A
SLC1A3
Pearson
0.925
0.900
0.875
0.850
0.825
0.800
0.775
0.750
0
100
200
300
400
Number of bins

Quantitative evaluation. a and b, The impact of ablating component focal loss on model performance. a, Comparison of accuracy in the first 15 epochs using focal loss and MSE loss for training. b, Cosine Similarity of predicted and correct results for models trained with focal loss and MSE loss. c-f, Effect of excluding diagonal counts on correlation coefficient. c, The proportion of diagonal count. d, The impact of removing diagonal count on correlation calculation. e, The proportion of zero counts. f, The impact of removing zero counts on correlation calculation. g, After training at different lengths, the similarity between the predicted/target pseudo-bulk Hi-C maps at the local regions.

Figure S4

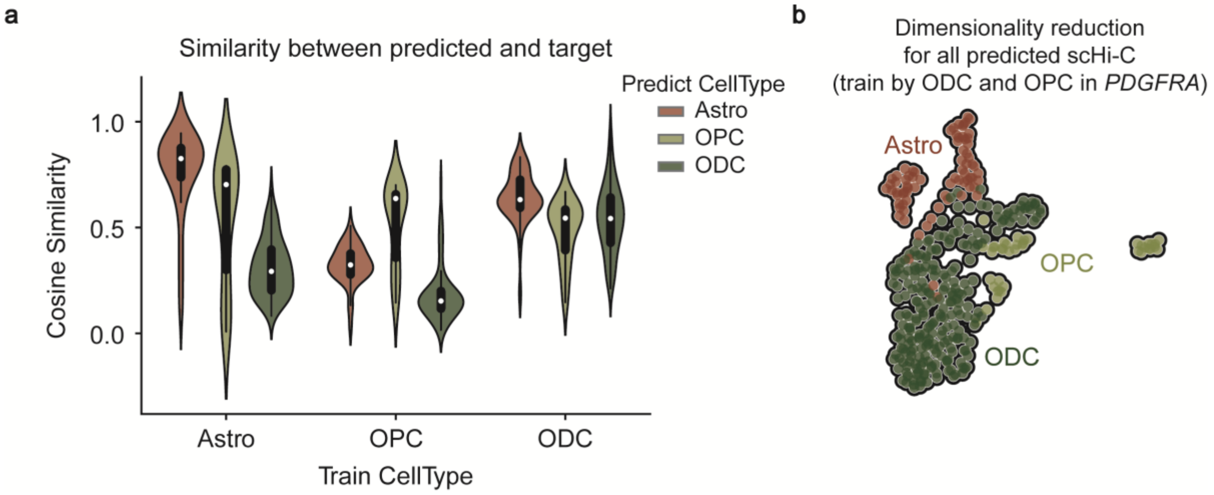

Examination of the type recognition and generalization. a, Three cell subtypes (Astro, ODC, and OPC) cross-validation comparison. Each violin shows the cosine similarity scores for target scHi-C maps and predicted scHi-C maps. b, First, use Principal Component Analysis (PCA) to reduce the features (interactions) of scHi-C maps to ten dimensions, then calculate a neighborhood graph of observations using the Scanpy::neighbors function, and finally use UMAP for visualization.

Figure S5

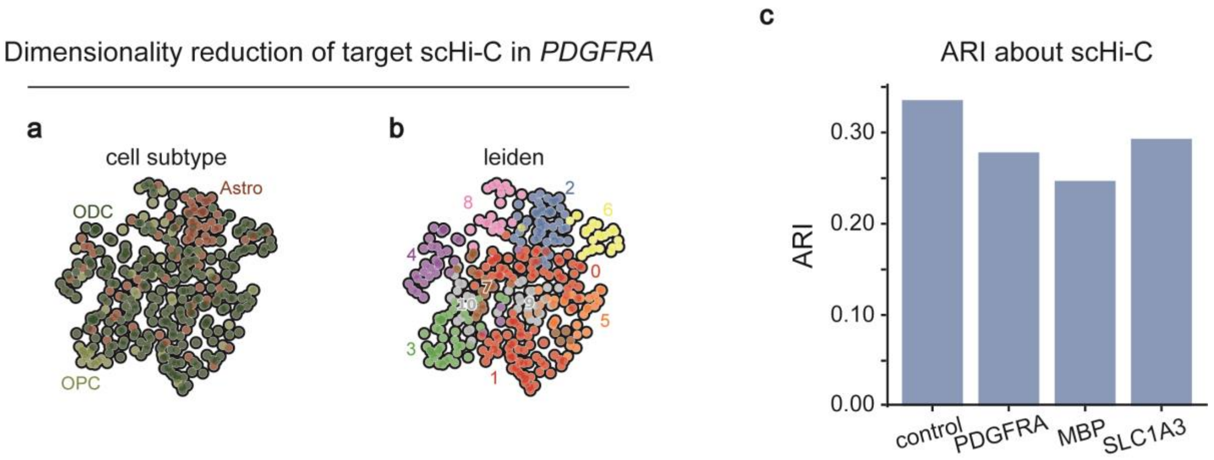

Reduced dimensional analysis. a and b, Reduced dimensional analysis of target scHi-C maps in *PDGFRA* locus. c, ARI coefficients for the background and three genes' prediction results.

Figure S6

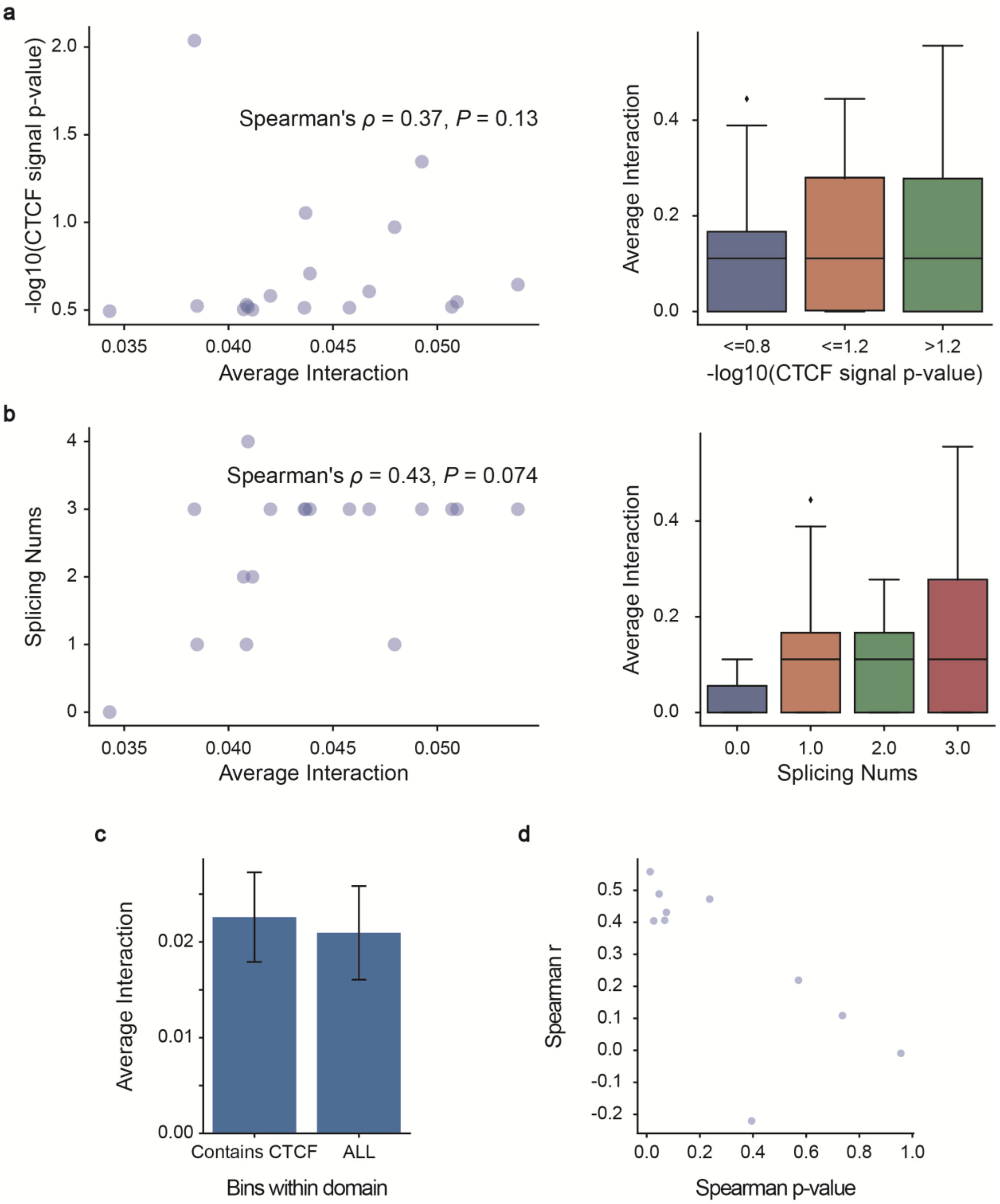

Association of CTCF/alternative splicing events with the interactions of predicted Hi-C maps. a and b, Left is the scatter plot of CTCF signal/number of candidate splicing events versus the

average interaction. Each dot corresponds to a bin of pseudo-bulk Hi-C map. Right is the average interaction at bins of scHi-C maps from different CTCF signal/number of candidate splicing events. c, Average interaction at bins of pseudo-bulk Hi-C maps from 12 random domains. d, Spearman correlation coefficient between the number of candidate splicing events and average interactions at 10 marker gene loci. The specific experimental design can be found in the methods.

Figure S7

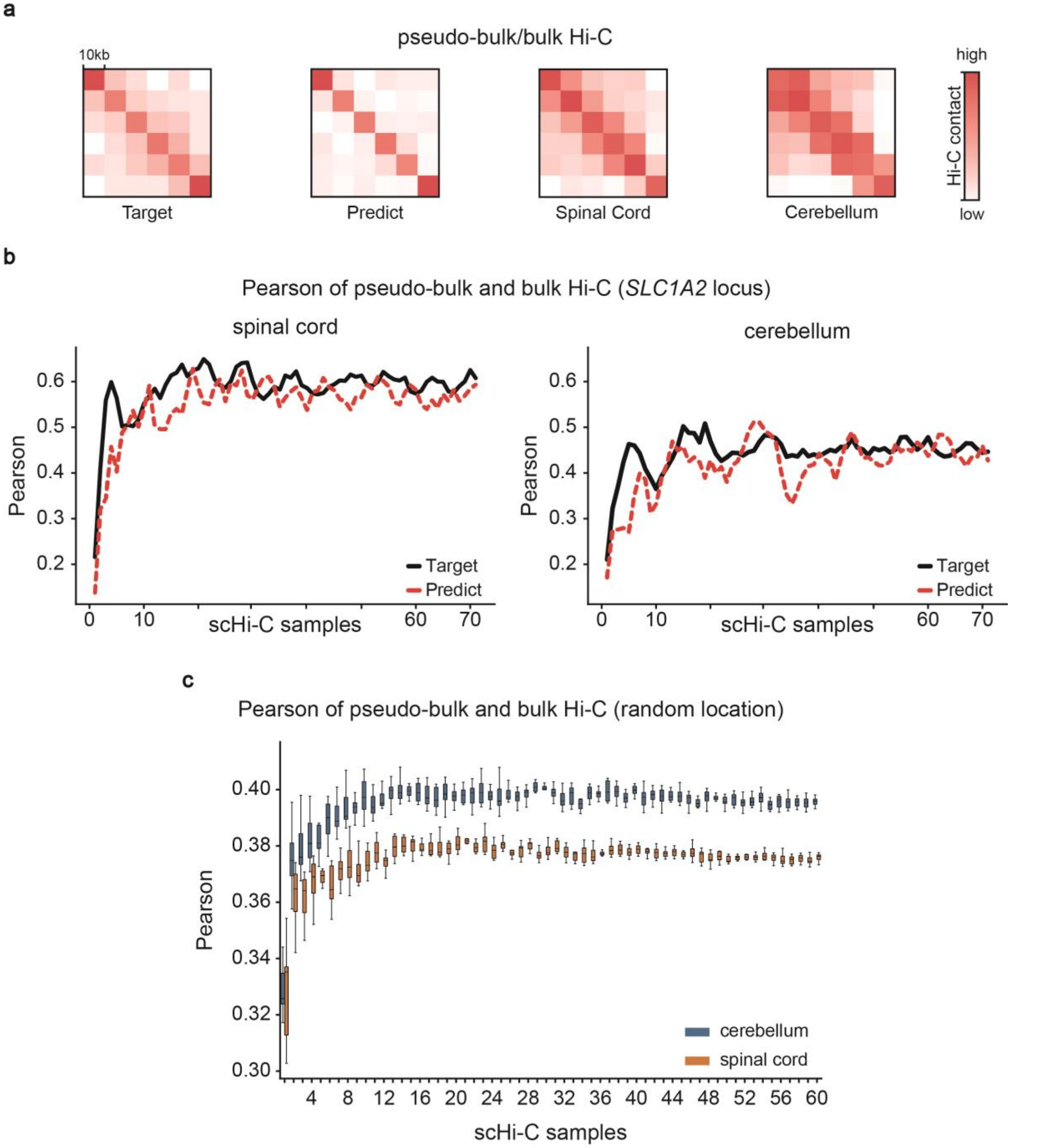

The similarity between predicted/target scHi-C maps of frontal cortex and the bulk Hi-C maps of two other sites (Spinal Cord and Cerebellum) within *SLC1A2* locus. a, Pseudo-bulk/Bulk Hi-C map of four objects (Target-pseudo-bulk, Predicted-pseudo-bulk, Spinal Cord-bulk, Cerebellum-bulk). b, With the increased input scHi-C samples, the Pearson value statistics of predicted/target pseudo-bulk Hi-C maps and spinal cord/cerebellum bulk Hi-C maps. c, The boxplot (n=10

random 1Mb locations) illustrates the similarity between the pseudo-bulk Hi-C maps and the bulk Hi-C maps.

Figure S8

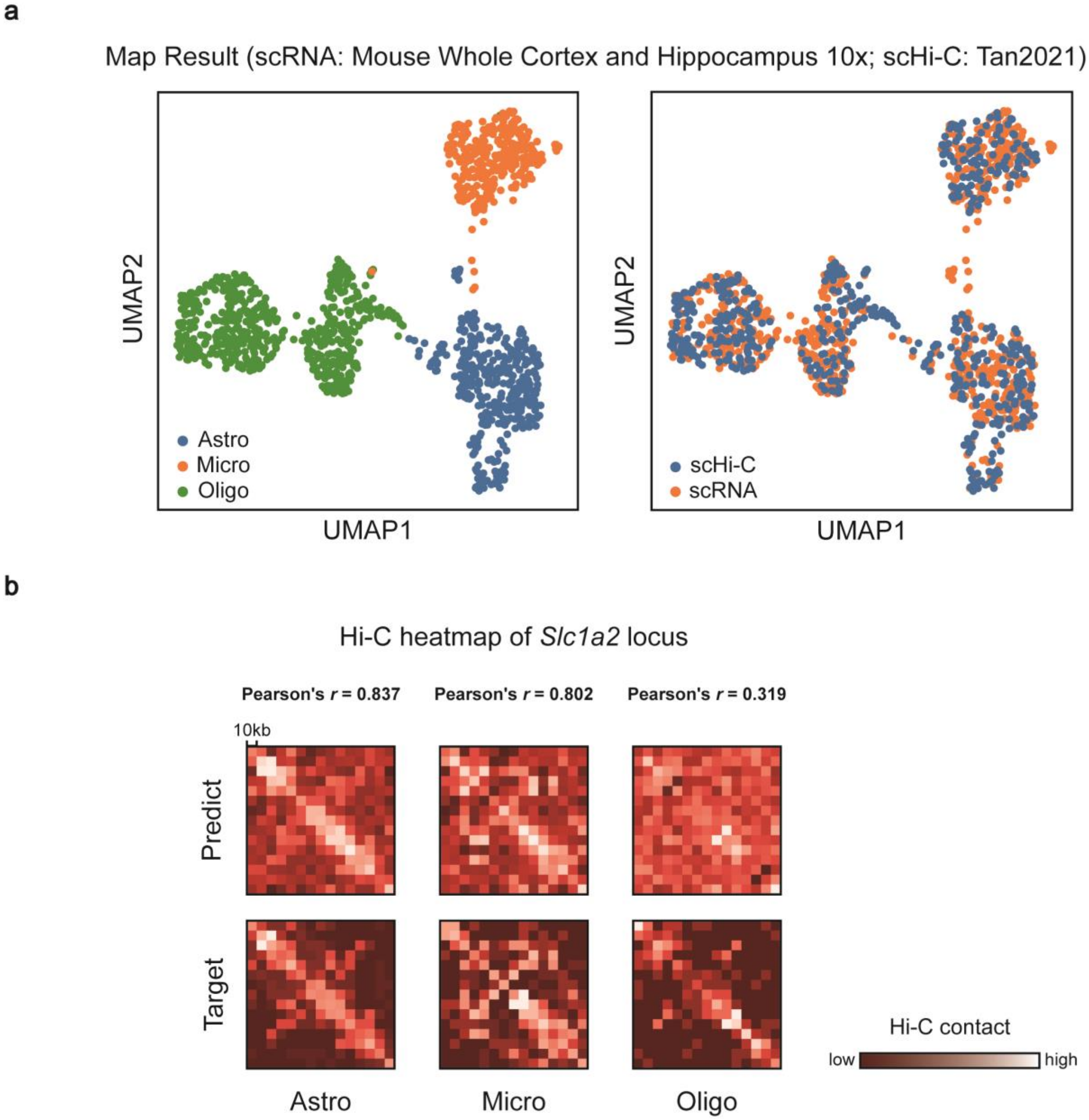

Examination of SEE's ability in the mouse dataset. a, Mapping with the same process on mouse-related scRNA (Mouse Whole Cortex and Hippocampus 10x) and scHi-C (Tan2021) datasets using the SEE method. After mapping, the merged data is dimensionally reduced and colored by cell subtype and data type. b, After a, the *Slc1a2* gene was used as the main subject to demonstrate the effect of SEE prediction under three cell subtypes(Astro, Micro, and Oligo).

Figure S9

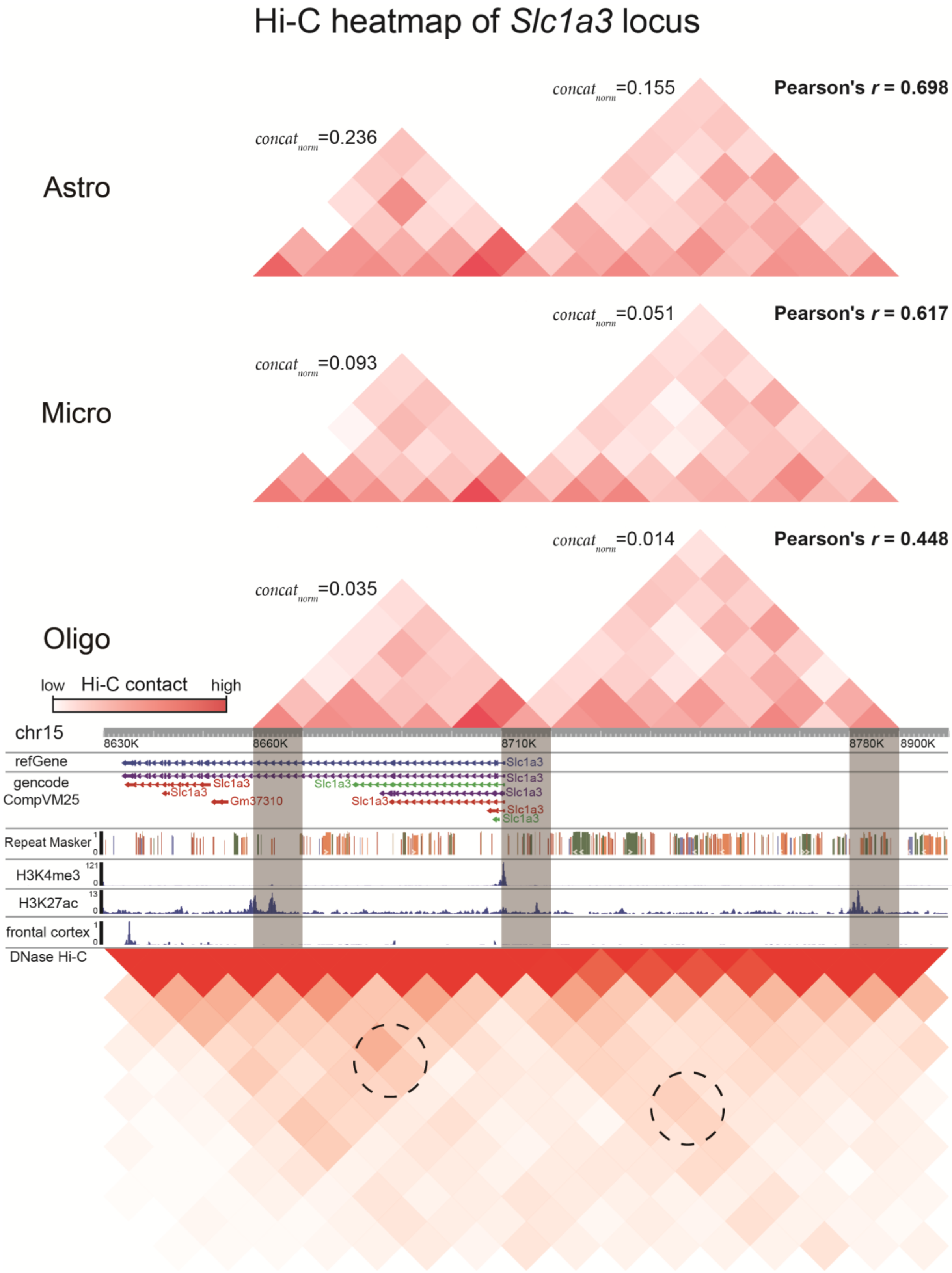
Hi-C heatmap of Slc1a3 locus
Astro
concat_norm=0.236
concat_norm=0.155
Pearson's r = 0.698
Micro
concat_norm=0.093
concat_norm=0.051
Pearson's r = 0.617
Oligo
concat_norm=0.035
concat_norm=0.014
Pearson's r = 0.448
low
Hi-C contact
high
chr15
8630K
8660K
8710K
8780K
8900K
refGene
gencode CompVM25
Slc1a3
Gm37310
Repeat Masker
H3K4me3
H3K27ac
frontal cortex
DNase Hi-C

By entering scRNA data with different characteristics, the results of SEE prediction show specific changes. The experiment was performed in mice brain cells with *Slc1a3* gene fragments (chr15: 8630,000-8800,000). scRNA data were divided into three categories by cell subtype (Astro, Micro, Oligo) and entered into the model separately, and then the data results were averaged.

Figure S10

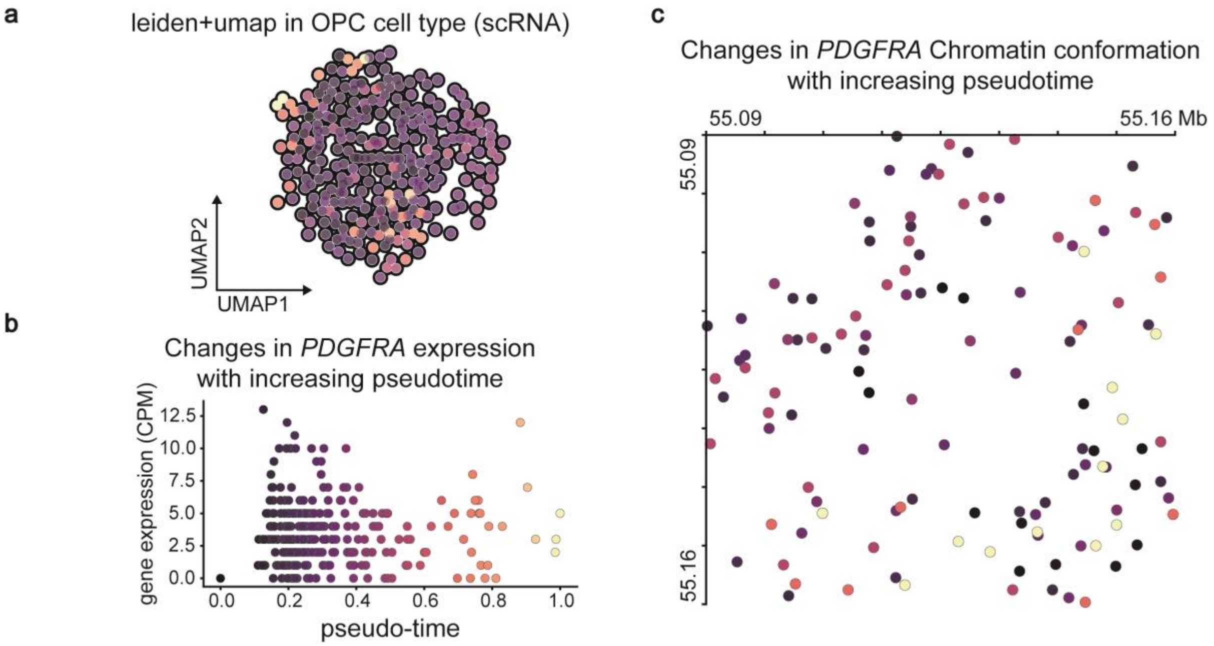

Simultaneous analysis of scRNA and scHi-C data on the pseudo-time scale. The coloring of this graph is unified: the closer to white, the greater the pseudo-time. a, UMAP projection of OPC-relate scRNA data colored by pseudo-time fraction calculated by Scanpy. b, Using the scRNA data used in a, calculate the association between *PDGFRA* gene expression and pseudo-time. c, Changes in *PDGFRA* chromatin conformation with increasing pseudo-time.

Figure S11

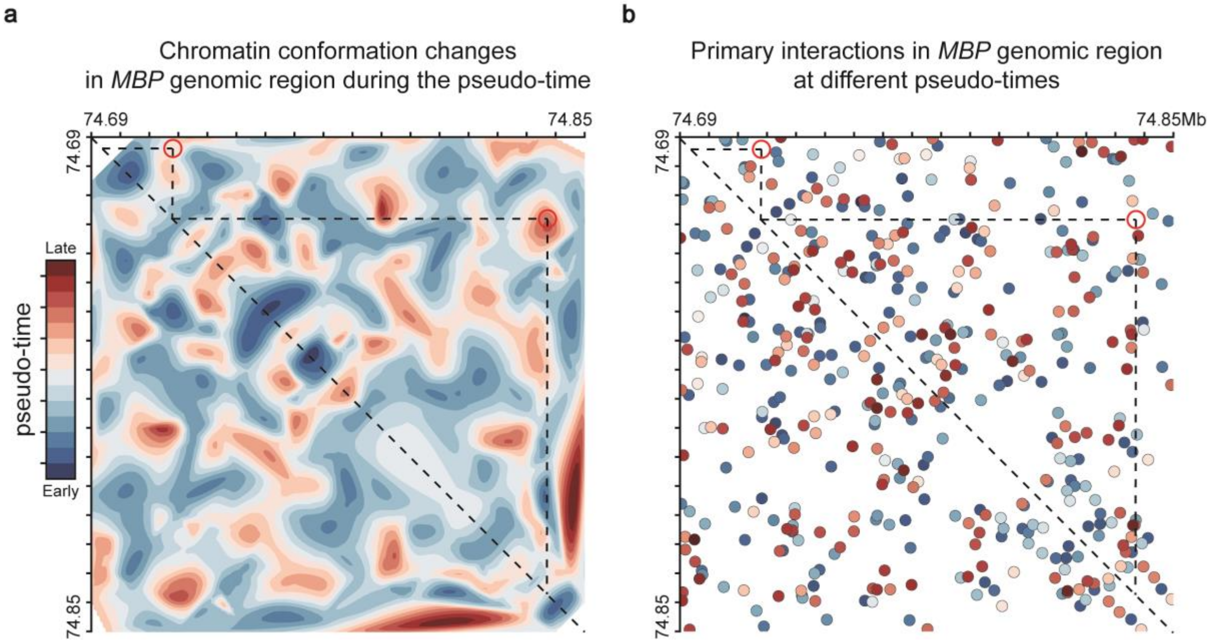

Pseudotemporal dynamics of chromatin conformation within the *MBP* locus. a, Calculated similarly to Figure 3c, but the scope is at the *MBP* locus. b, All coordinate values calculated in Figure 3c are plotted in the same graph and colored using pseudo-time.

Figure S12

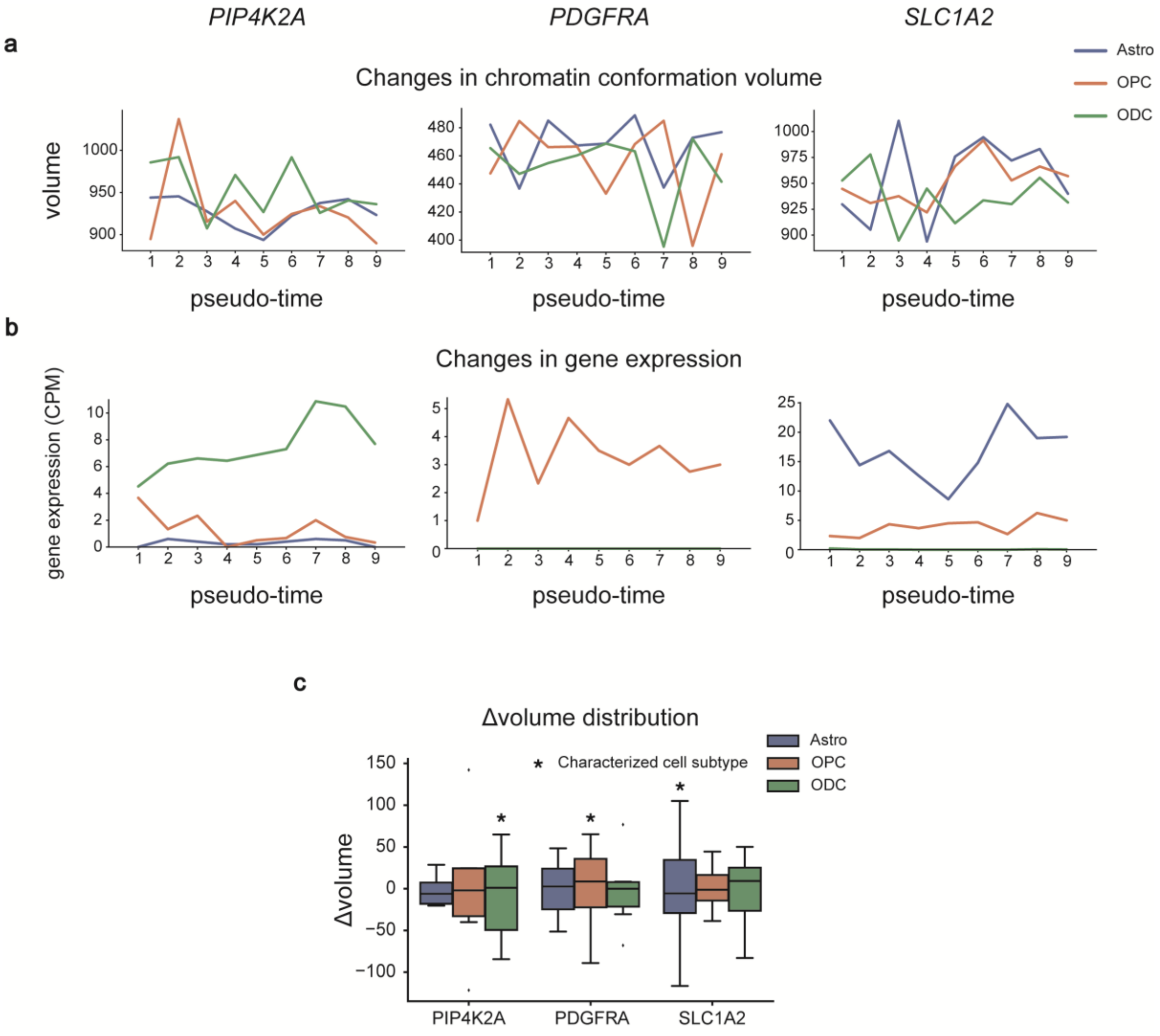

The dynamic features of DEGs in characterized/uncharacterized cell subtypes. a, The chromatin conformation volume changes over pseudo-time. The volume is calculated using 3D coordinates derived from 3DMax. b, The expression count (CPM) changes over pseudo-time. c, The distribution of Δvolume under different genes and cell subtypes.

Figure S13

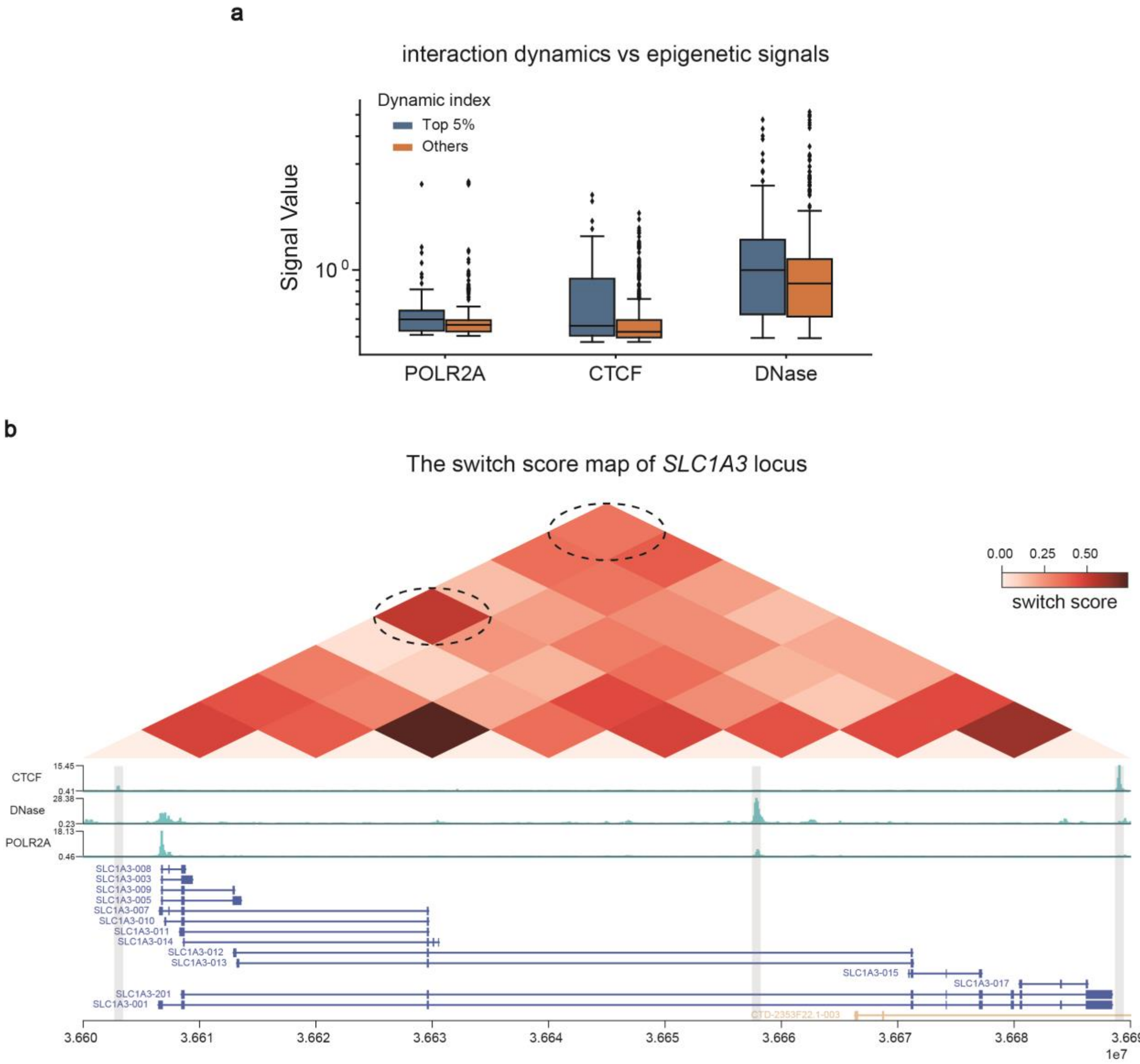

Association of epigenetic signals with the interaction dynamics. a, The boxplot depicts the correlation between dynamic levels and POLR2A, CTCF, and DNase signal values. b, The switch score map of *SLC1A3* locus in Astro cell subtype.

Figure S14

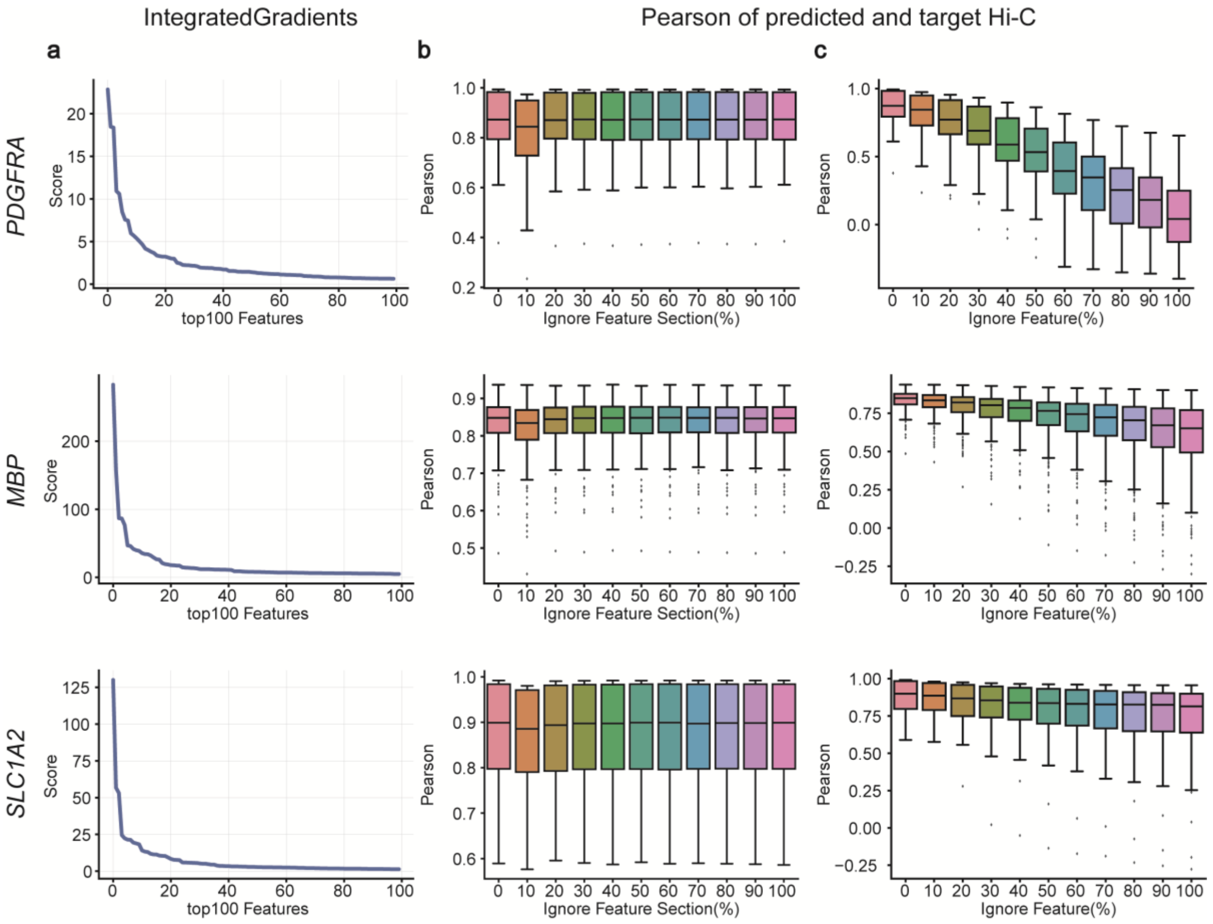

Effect of features (chromatin regulators) on the model. a, IntegratedGradients score of the first 100 important features. b, Pearson correlation coefficient between model predictions and the target data after setting n~(n+10)% of the top 100 features to zero. c, Pearson correlation coefficient between model predictions and target data after setting the top n% of the top 100 features to zero.

Figure S15

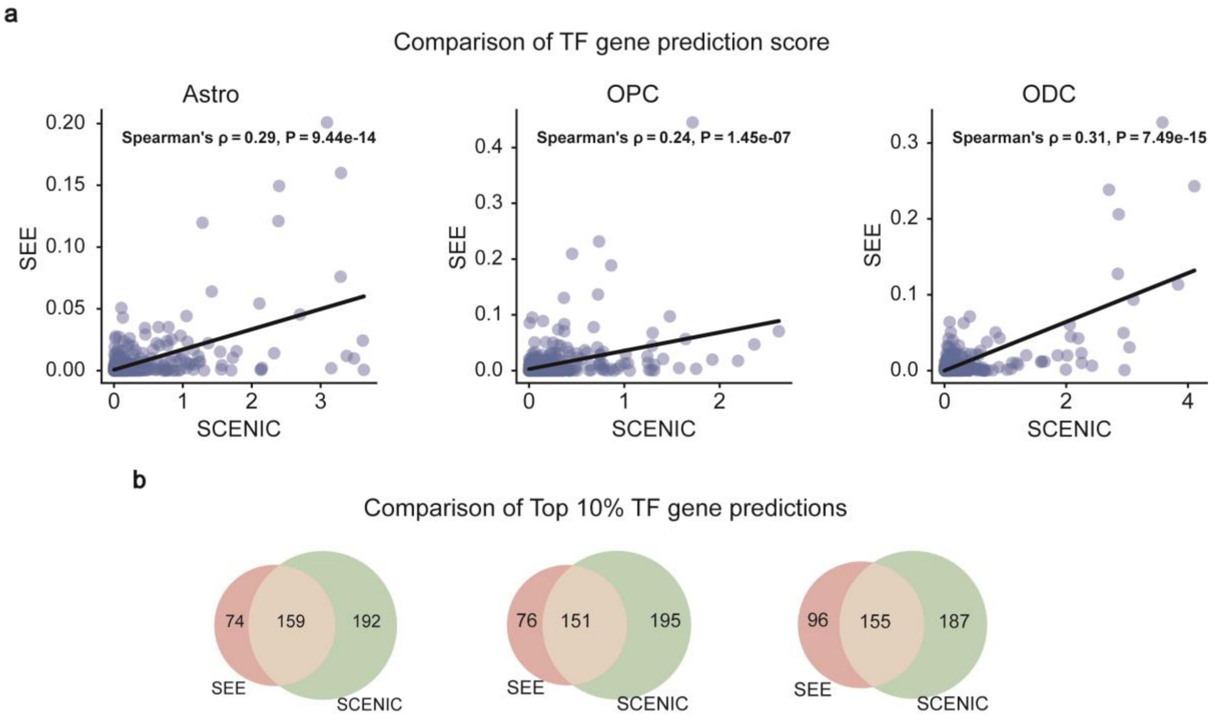

Comparative experiment with SCENIC on TF prediction. a, Correlation analysis of the significant TFs obtained by SEE with the results of SCENIC. We chose three marker genes to represent each cell subtype separately (Astro uses *SLC1A2*, *SLC1A3*, and *GPM6A*; OPC uses *PDGFRA*, *PTPRZ1*, and *VCAN*; ODC uses *MBP*, *QKI*, and *ENPP2*). For SCENIC, the calculation is summarized as follows: the scRNA data is first preprocessed with the filter_genes function, and then the TF gene score is calculated using the grnboost2 method. (Methods) b, Venn diagram on the important TF factors predicted by SEE and SCENIC.

Figure S16

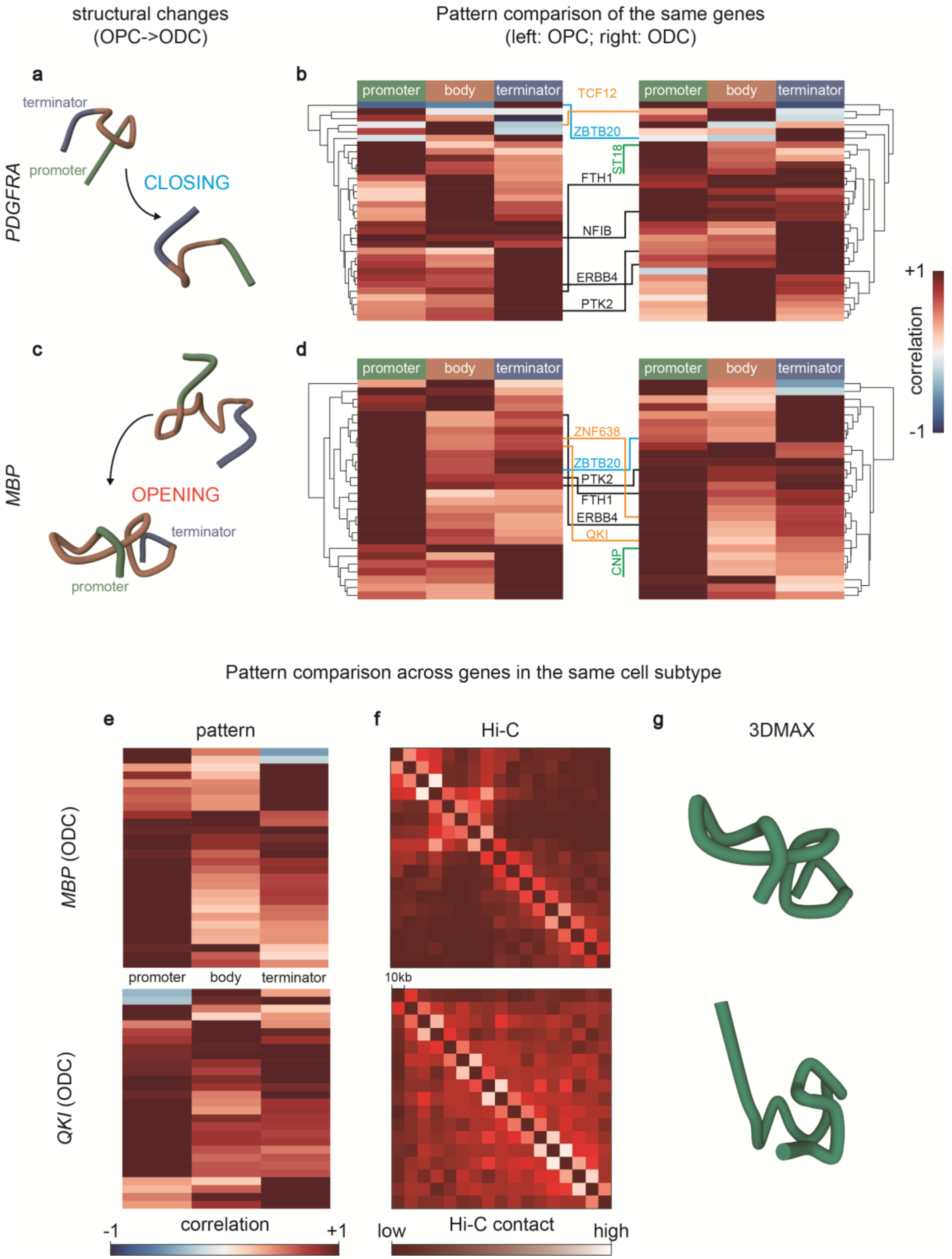

Validation of chromatin regulators affecting interaction dynamics. a and c, 3D structural changes of *PDGFRA* (a) / *MBP* (c) locus from OPC to ODC. b and d, The regulatory pattern differs

between different cell subtypes for the same genomic regions (*PDGFRA* (b), and *MBP* (d)). Each heatmap consists of 20 chromatin regulators that differ significantly at the target gene's three positions (promoter, body, and terminator). Colors indicate the importance score of chromatin regulators calculated by IntegratedGradients (Methods). The blue line (*ZBTB20*) indicates factors that repress gene transcription; the orange lines (*TCF12*, *ZNF638*, and *QKI*) indicate factors that promote gene transcription; the green lines (*ST18* and *CNF*) indicate factors that appear only in single-cell subtype; the remaining lines (black) are fundamental transcription factors. (Methods) e, Expression patterns exhibited by gene promoter, body, and terminator. f, Pseudo-bulk Hi-C maps in *MBP*/*QKI* locus. g, The 3D structure of *MBP*/*QKI* locus (predicted by 3DMAX).

Figure S17

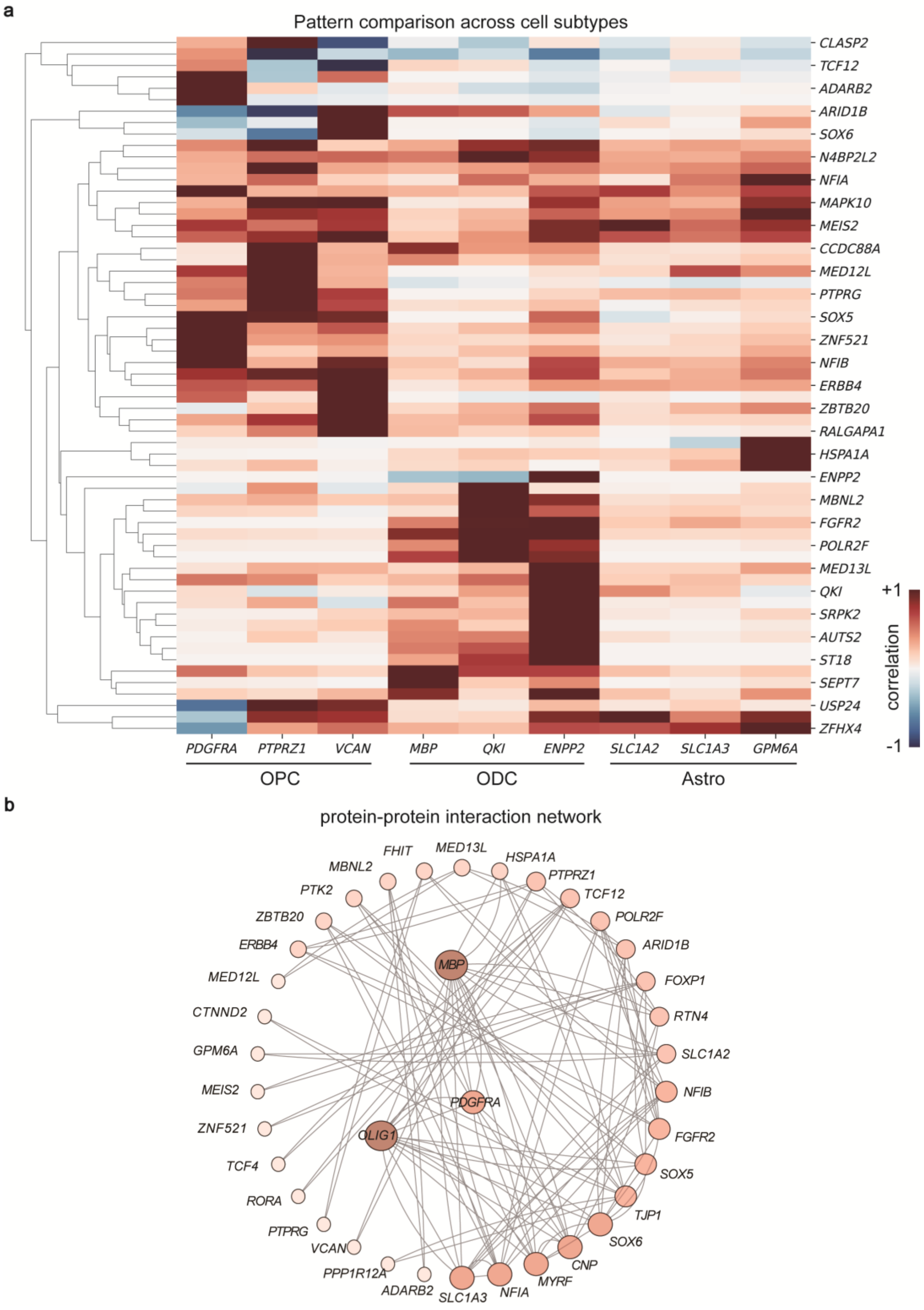
a
Pattern comparison across cell subtypes
CLASP2
TCF12
ADARB2
ARID1B
SOX6
N4BP2L2
NFIA
MAPK10
MEIS2
CCDC88A
MED12L
PTPRG
SOX5
ZNF521
NFIB
ERBB4
ZBTB20
RALGAPA1
HSPA1A
ENPP2
MBNL2
FGFR2
POLR2F
MED13L
QKI
SRPK2
AUTS2
ST18
SEPT7
USP24
ZFHX4
PDGFRA
PTPRZ1
VCAN
MBP
QKI
ENPP2
SLC1A2
SLC1A3
GPM6A
OPC
ODC
Astro
+1
correlation
-1
b
protein-protein interaction network
FHIT
MED13L
HSPA1A
MBNL2
PTPRZ1
PTK2
TCF12
ZBTB20
POLR2F
ERBB4
ARID1B
MED12L
MBP
FOXP1
CTNND2
RTN4
GPM6A
SLC1A2
MEIS2
NFIB
PDGFRA
ZNF521
OLIG1
FGFR2
TCF4
SOX5
RORA
TJP1
PTPRG
SOX6
VCAN
CNP
PPP1R12A
MYRF
ADARB2
SLC1A3
NFIA

Analysis of the regulatory patterns of representative genes in different cell subtypes. a, Three marker genes were selected for each cell subtype (Astro, OPC, ODC). Chromatin regulators were calculated for each marker gene. Then, the top 20 chromatin regulators with the greatest correlation differences were found within these nine gene ranges, and the heatmap enrichment was calculated. b, Network diagram of protein-protein interaction (PPI)[1] containing marker gene and chromatin regulators used in a. Individual genes not connected to most genes have been removed.

Figure S18

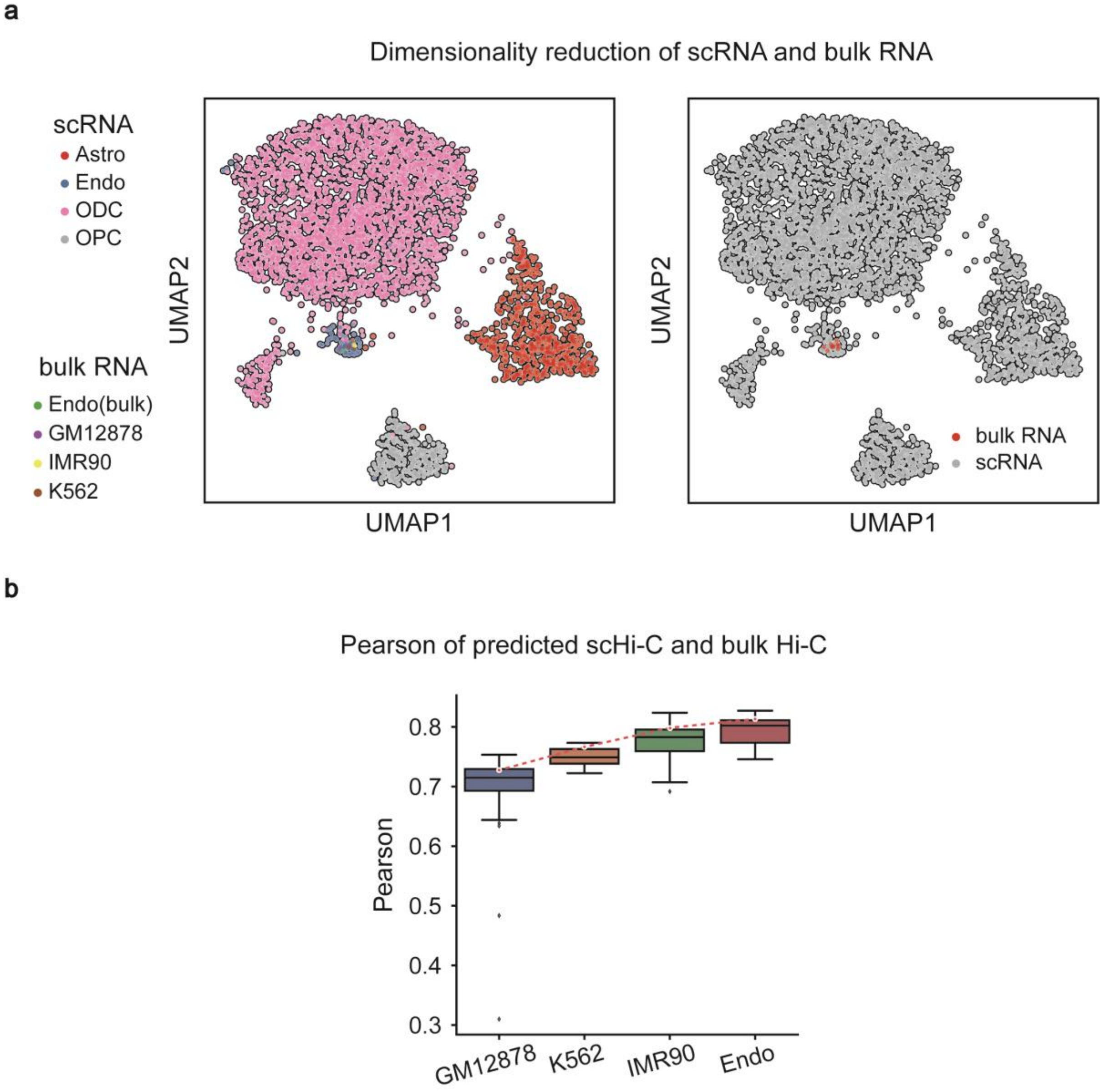

Experiment on untrained bulk data. a, Mapping bulk RNA data (Endo (bulk), GM12878, IMR90, and K562) to the space of learned scRNAs (human). b, Pearson scores for predicted scHi-C maps and bulk Hi-C maps. scHi-C map was obtained by transferring the tag of bulk RNA data to scRNA data using scanorama after a, and then using SEE to predict the scRNA data that can represent the bulk tag.

Figure S19

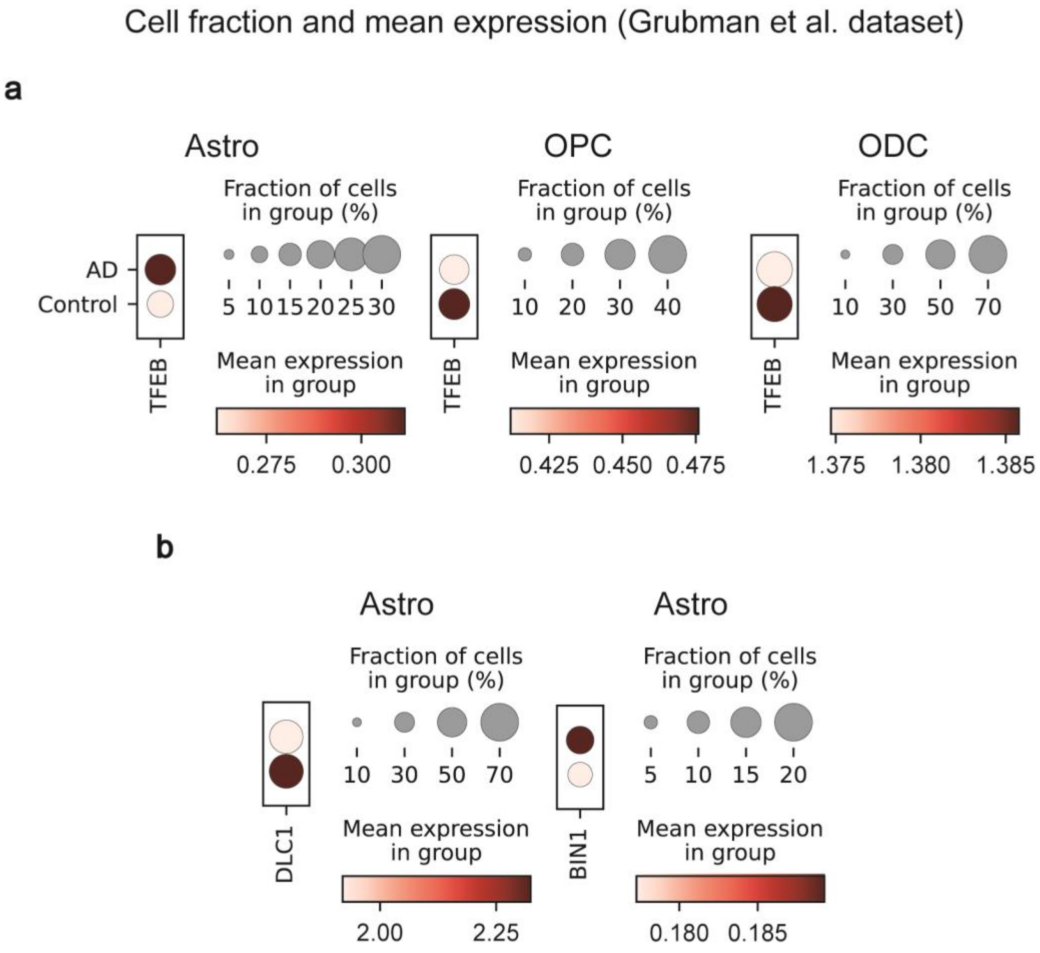

Quality check of Alzheimer's disease-related dataset. a, Cell fraction and mean expression of *TFEB* in Astro, OPC, and ODC. b, Cell fraction and mean expression of *DLC1* and *BIN1* in Astro.

Figure S20

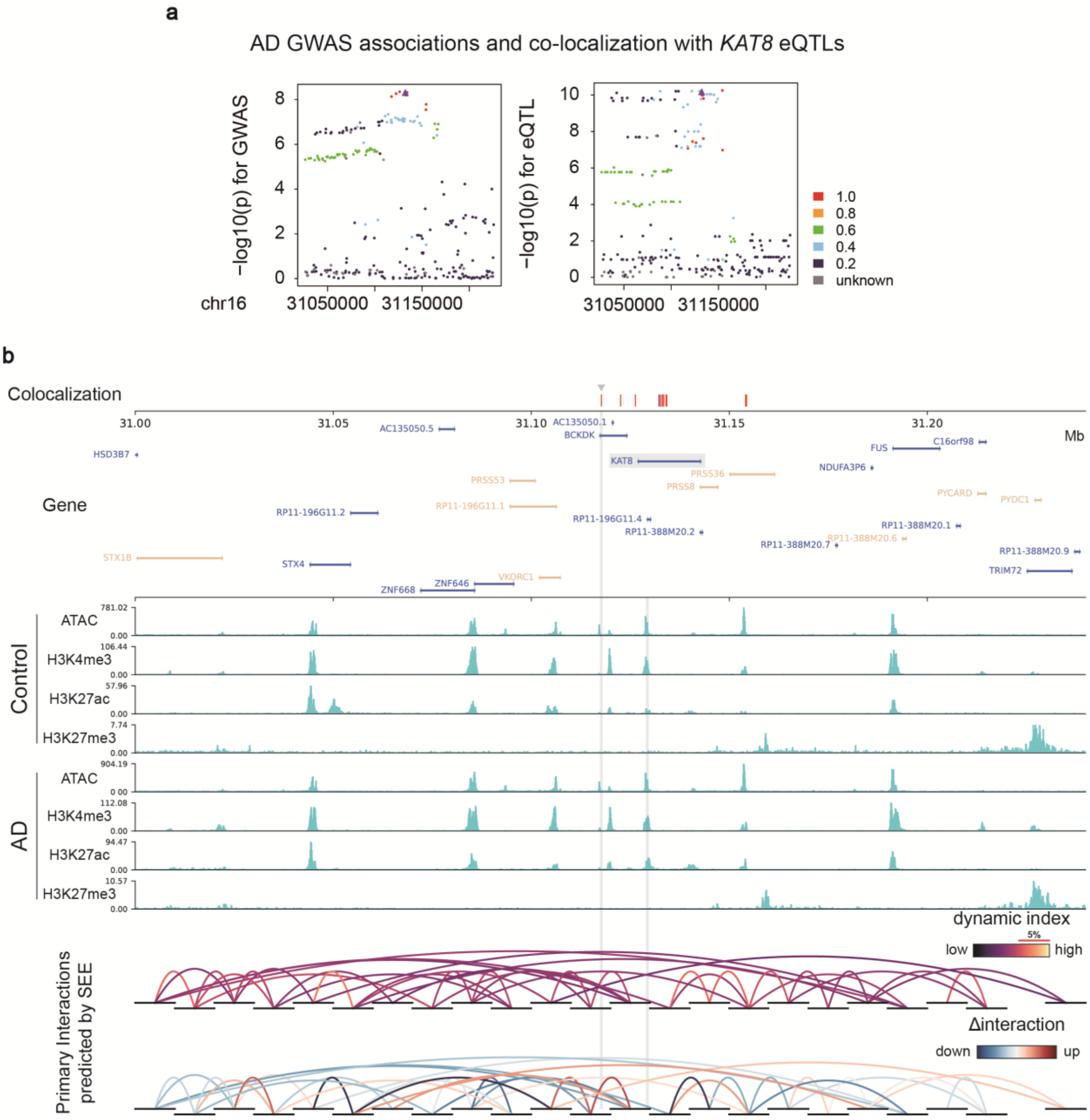

Joint analysis of SEE and GWAS co-localization at *KAT8* locus. a, Plot of locus around *KAT8* gene. The AD GWAS associations (left) were colocalized with *KAT8* eQTLs in the prefrontal cortex (right). The y-axis showed -log10P of associations in both plots. Colors indicated linkage disequilibrium (LD) r2 of each variant relative to the colocalized variant rs11865499 labeled in a purple triangle. b, Similar to Figure 6c, but analyzed for the *KAT8* locus.

Video S1

Joint analysis of primary interactions, 3D structures, volumes, and gene expression in the *QKI* locus.

Supplementary References

[1] P. Shannon, A. Markiel, O. Ozier, N. S. Baliga, J. T. Wang, D. Ramage, N. Amin, B. Schwikowski, T. Ideker, *Genome Res* **2003**, *13*, 2498.
